# Exploring Advanced CRISPR Delivery Technologies for Therapeutic Genome Editing

**DOI:** 10.1002/smsc.202400192

**Published:** 2024-07-25

**Authors:** Neda Rostami, Mohammad Mahmoudi Gomari, Edris Choupani, Shadi Abkhiz, Mahmood Fadaie, Seyed Sadegh Eslami, Zahra Mahmoudi, Yapei Zhang, Madhu Puri, Fatemeh Nafe Monfared, Elena Demireva, Vladimir N. Uversky, Bryan Ronain Smith, Sidi A. Bencherif

**Affiliations:** ^1^ Department of Chemical Engineering Arak University Arak 3848177584 Iran; ^2^ Department of Medical Biotechnology Faculty of Allied Medicine Iran University of Medical Sciences Tehran 1449614535 Iran; ^3^ Department of Molecular Biology and Biochemistry Rutgers University Piscataway NJ 08854 USA; ^4^ Department of Genetics and Molecular Biology School of Medicine Isfahan University of Medical Sciences Isfahan 8174673461 Iran; ^5^ Molecular Proteomics Laboratory Baker Heart and Diabetes Institute Melbourne Victoria 3004 Australia; ^6^ Department of Biomedical Engineering Institute for Quantitative Health Science and Engineering Michigan State University East Lansing MI 48824 USA; ^7^ Department of Virology School of Public Health Tehran University of Medical Sciences Tehran 1416634793 Iran; ^8^ Department of Molecular Medicine and USF Health Byrd Alzheimer's Research Institute Morsani College of Medicine University of South Florida Tampa FL 33612 USA; ^9^ Department of Radiology Stanford University Stanford CA 94305 USA; ^10^ Departments of Chemical Engineering and Bioengineering Northeastern University Boston MA 02115 USA; ^11^ Harvard John A. Paulson School of Engineering and Applied Sciences Harvard University Cambridge MA 02138 USA; ^12^ Biomechanics and Bioengineering Sorbonne University, UTC CNRS UMR 7338, University of Technology of Compiègne 60203 Compiègne France

**Keywords:** biomaterials, clustered regularly interspaced short palindromic repeat, exosomes, genome editing, nanoparticles

## Abstract

The genetic material within cells plays a pivotal role in shaping the structure and function of living organisms. Manipulating an organism's genome to correct inherited abnormalities or introduce new traits holds great promise. Genetic engineering techniques offers promising pathways for precisely altering cellular genetics. Among these methodologies, clustered regularly interspaced short palindromic repeat (CRISPR), honored with the 2020 Nobel Prize in Chemistry, has garnered significant attention for its precision in editing genomes. However, the CRISPR system faces challenges when applied in vivo, including low delivery efficiency, off‐target effects, and instability. To address these challenges, innovative technologies for targeted and precise delivery of CRISPR have emerged. Engineered carrier platforms represent a substantial advancement, improving stability, precision, and reducing the side effects associated with genome editing. These platforms facilitate efficient local and systemic genome engineering of various tissues and cells, including immune cells. This review explores recent advances, benefits, and challenges of CRISPR‐based genome editing delivery. It examines various carriers including nanocarriers (polymeric, lipid‐derived, metallic, and bionanoparticles), viral particles, virus‐like particles, and exosomes, providing insights into their clinical utility and future prospects.

## Graphical abstract

This review examines the potential of clustered regularly interspaced short palindromic repeat (CRISPR) technology in cellular genetics, focusing on challenges like low delivery efficiency and off‐target effects. It discusses innovative carriers—viral, lipid‐based, polymeric particles, and exosomes—that improve stability, reduce side effects, and enhance editing capabilities, aiming to optimize CRISPR delivery for therapeutic use.

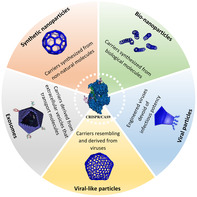

## 1 Introduction

The ability to accurately and purposefully edit any part of an organism's genome to treat diseases and eliminate hereditary defects has long been a scientific dream.^[^ ^1^ ^]^ The recent significant advances made in the field of gene therapy approaches have improved the success rates of these methods, which promise to cure a variety of human defects and diseases. Various forms of gene editing are being exploited as potential approaches to treat many diseases, including genetic disorders and some types of cancer.^[^ ^2^, ^3^, ^4^ ^]^

Early gene therapy technologies relied on the use of viral and nonviral vector delivery of DNA transgenes in vivo or ex vivo, designed to compensate for a missing or defective gene product or express a proapoptotic gene in cancer cells.^[^ ^5^, ^6^ ^]^ Subsequently, precise genome editing technologies were developed based on the use of site‐specific or designer nucleases.^[^ ^7^, ^8^ ^]^ Conventional site‐specific genome editing typically involves the introduction of a double‐strand break (DSB) at a target DNA site and its subsequent repair by the endogenous DNA repair machinery, leading to the correction of mutations or the removal or insertion of specific DNA sequences.^[^ ^9^ ^]^ Site‐specific nuclease technologies, including transcription activator‐like effector nucleases (TALENs) and zinc finger nucleases (ZFNs), have seen limited use and adoption despite their numerous advantages. This limitation primarily arises from their complexity, variability in efficiency depending on the target sequence, high cost, engineering difficulty of protein hybrids, lack of multiplexing potential, and high off‐target activity.^[^ ^10^, ^11^, ^12^ ^]^

One site‐specific nuclease technology that has deservedly attracted much recent attention is clustered regularly interspaced short palindromic repeats (CRISPRs) and CRISPR‐associated protein (Cas), collectively referred to as CRISPR/Cas. It is a versatile strategy for manipulating the genomes of diverse organisms.^[^ ^13^, ^14^ ^]^ Deciphering how the complex functions were achieved once a reconstituted system of Cas9 protein and CRISPR RNA (crRNA) was demonstrated to facilitate DNA cleavage in vitro.^[^ ^15^, ^16^ ^]^ Key advantages of this system, compared with predecessor designer nucleases such as ZFNs and TALENs, include simple programmability, multiplex targeting of distinct gene loci directed by multiple gRNAs, cost‐effectiveness, high controllability, simplicity of design, accuracy, ease of use, longer half‐life, and suitability for various applications, including gene therapy.^[^ ^17^, ^18^, ^19^ ^]^

Seminal studies adapting the CRISPR/Cas9 system from *Streptococcus* *pyogenes* for genome editing in human and mouse cells^[^ ^20^, ^21^, ^22^ ^]^ revolutionized the field of genome engineering. They demonstrated that a codon‐optimized CRISPR‐associated 9 (Cas9) protein and a single guide RNA (sgRNA, henceforth referred to as gRNA) in a reduced format are sufficient to cause DSBs in mammalian cells. Extensive efforts were subsequently made to edit the genomes of eukaryotic and prokaryotic cells,^[^ ^23^, ^24^ ^]^ turning CRISPR into a versatile and powerful approach for genome editing in a variety of living organisms.^[^ ^25^, ^26^, ^27^ ^]^ The scientific community has achieved considerable advances in editing efficiency and precision because CRISPR was first discovered and adapted for use in eukaryotic cells.^[^ ^28^ ^]^ The first clinical trial for human genome manipulation was conducted in 2016 at the West China Hospital, where the T‐cell genome was engineered ex vivo to increase its potency against cancer cells,^[^ ^29^ ^]^ and currently, many human trials using CRISPR therapeutics are active on ClinicalTrials.gov. As more CRISPR‐based therapeutics become ready for clinical applications, one critical aspect of their success is the specific and efficient delivery of the therapeutic and the amelioration of undesired side effects or toxicity.^[^ ^30^ ^]^

The CRISPR system has three main formats of delivery to the target site without the use of viral vectors: 1) delivery of a plasmid DNA encoding both the Cas protein and gRNA, 2) delivery of the elements as RNA in the form of gRNA plus mRNA that can be converted into Cas nucleases through cellular translation in the cytoplasm, and 3) delivery of a ribonucleoprotein (RNP) complex consisting of Cas protein and gRNA. RNP delivery allows the CRISPR complex to act with the fastest and most transient expression kinetics, which is thought to increase the safety of genome editing by reducing potential off‐target effects due to random DNA insertion or prolonged Cas9 expression.^[^ ^31^ ^]^ If precise genome editing is required, then a DNA‐based donor must be codelivered with the CRISPR components to serve as a template during the DNA repair process. To maintain low off‐target effects and high efficiency, reliable and specific delivery of CRISPR to desired target tissues and cells is critical. The use of engineered carriers is an efficient way to improve the performance of CRISPR‐based genome editing platforms.^[^ ^32^ ^]^

The development of engineered carriers has revolutionized disease treatment. These carriers enhance the safety, efficiency, and specificity of CRISPR‐based therapies by optimizing their capacity and pharmacokinetics.^[^ ^33^, ^34^ ^]^ Further functionalization and engineering efforts can improve their effectiveness.^[^ ^35^ ^]^ Additionally, these delivery platforms can minimize off‐target effects associated with CRISPR‐based treatments. By enhancing the precision and delivery of gene‐editing tools, these advancements hold significant promise for personalized cancer therapies and other genetic disorders.^[^ ^36^, ^37^ ^]^ For example, the liver‐targeting gene‐hybridizing‐tyrosine kinase inhibitor fusogenic liposome has been designed to overcome epidermal growth factor receptor‐mediated drug resistance in hepatocellular carcinoma.^[^ ^38^ ^]^

**Figure 1:**
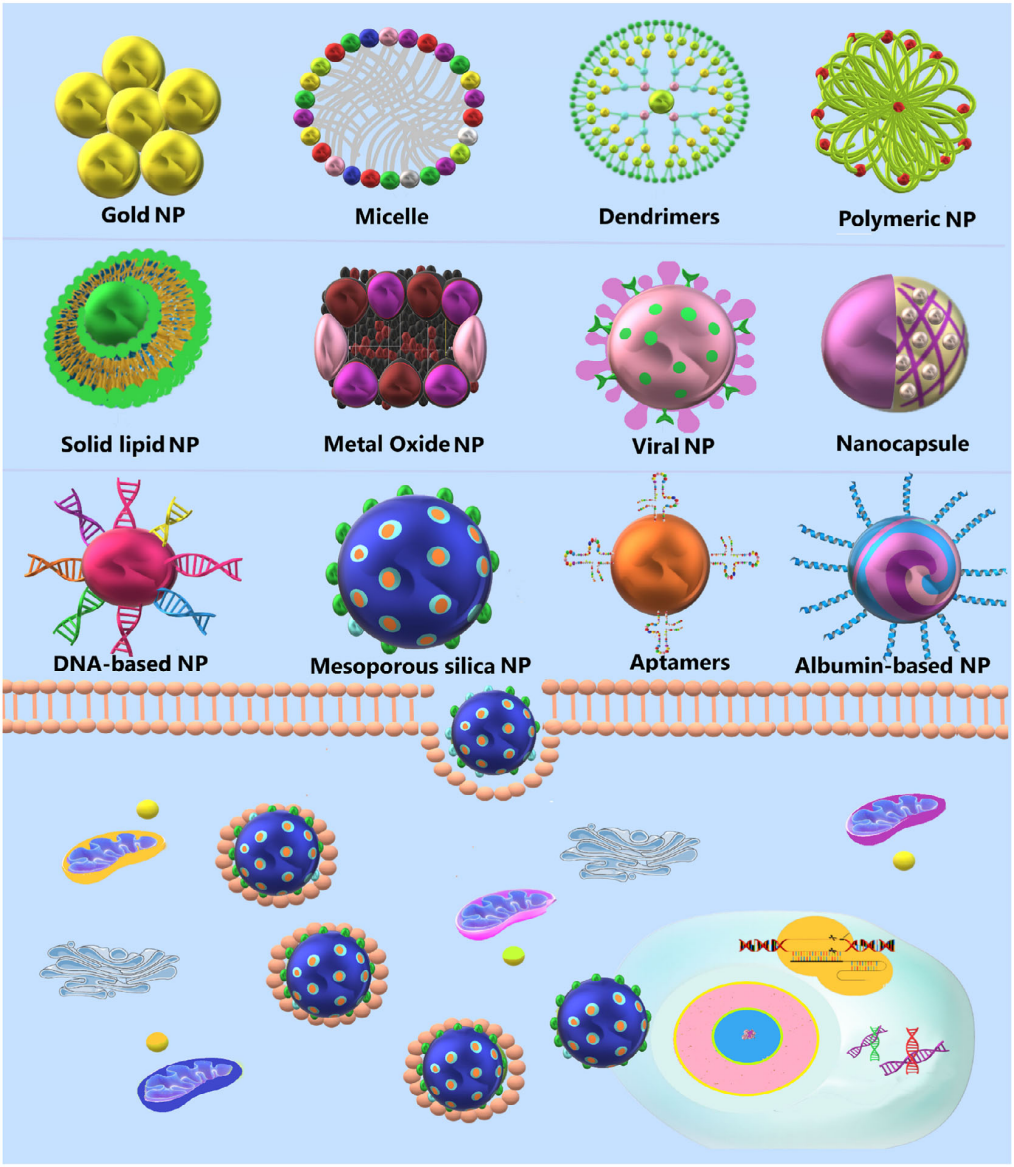
Different classes of engineered carriers are used for CRISPR delivery. A well‐designed delivery system can improve CRISPR efficiency by facilitating its membrane penetration and entry into the cells.

So far, a wide range of nanomaterials, including bionanocarriers, metal nanocarriers, polymeric nanoparticles (NPs), and hybrid NPs (produced from combinations of different materials such as organic and inorganic materials) (**Figure** **1**), along with other engineered carriers such as viral particles, virus‐like particles (VLPs), and exosomes, have been developed for the delivery of CRISPR.^[^ ^39^, ^40^, ^41^, ^42^, ^43^, ^44^ ^]^ There are several reasons to use engineered particles as safe, effective carriers of CRISPR components to cells and tissues both ex vivo and/or in vivo, including: 1) protecting loaded cargo from destruction until it reaches the delivery site, 2) high efficiency for targeting diseases by binding specific types of cells or tissues, and 3) ability to deliver large cargo including RNP and large, multidomain proteins.^[^ ^45^ ^]^

The tools used to deliver CRISPR for gene editing can be classified into three primary groups: viral vectors, nonviral vectors, and physical delivery methods.^[^ ^46^ ^]^ While viral vectors and some nonviral methods are commonly used for in vivo applications, physical delivery methods such as electroporation, microinjection, and hydrodynamic injection are predominantly used in vitro. These methods provide direct and efficient means of introducing RNA, DNA, or RNP CRISPR components into cells.^[^ ^46^ ^]^ Microinjection, for example, has been extensively employed in generating transgenic animals by injecting CRISPR into zygotes or early embryos, followed by transferring them into the oviducts or uteri of surrogate female animals. It allows for precise and highly efficient delivery (≈100%), but is labor‐intensive and not suitable for high‐throughput or most clinical applications.^[^ ^46^, ^47^ ^]^

Electroporation, which uses electrical pulses to permeabilize cell membranes, is effective for a wider range of cell types than other delivery techniques, but it can result in high cell mortality and is not suitable for in vivo applications.^[^ ^48^ ^]^ Hydrodynamic injection involves the rapid injection of a large volume of solution (8–10% of body weight) containing gene editing cargo into the bloodstream of an animal. The resulting pressure in in the vessels allows the cargo to pass into cells by temporarily enhancing permeability into endothelial and parenchymal cells.^[^ ^49^ ^]^ However, hydrodynamic delivery is not currently being considered for clinical applications due to potential physiological complications, including cardiac dysfunction, elevated blood pressure, and liver expansion.^[^ ^46^, ^49^ ^]^

Viral vectors are among the most commonly used delivery tools for CRISPR gene editing in translational research and clinical development. Adeno‐associated viruses (AAVs) are favored for their low immunogenicity, low cytotoxicity, and limited integration into the host cells.^[^ ^50^ ^]^ However, their limited packaging capacity restricts the size of the genetic material they can deliver; AAVs can carry only smaller genetic cargo of less than ≈5 kb.^[^ ^51^ ^]^ Therefore, developing complementary or alternative strategies that address AAV cargo limits is necessary. Key approaches include use of compact Cas orthologs for single AAV vectors or dual AAV vectors with split CRISPR components across two vectors.^[^ ^52^ ^]^ Another approach is to use AAVs to deliver sgRNA into cells that have already been altered to express the Cas9 protein.^[^ ^50^ ^]^ Lentiviral vectors, another popular choice, have a greater cargo capacity (8–10 kb) and can integrate into the host genome, allowing for long‐term expression of CRISPR components. This makes them particularly useful for efficiently transducing nondividing and terminally differentiated cells.^[^ ^53^ ^]^ However, lentiviral vectors can lead to insertional mutagenesis.^[^ ^54^ ^]^ Adenoviruses are another type of viral vector capable of carrying larger genetic payloads (up to 36 kb), providing high transduction efficiency and reducing the risk of insertional mutagenesis and oncogenicity observed with other viral vectors.^[^ ^55^ ^]^ However, adenoviral vectors also pose a higher risk of eliciting immune responses, which can complicate their use for therapeutic applications.^[^ ^56^ ^]^

Nonviral particles offer an increasingly important alternative to viral delivery systems. VLPs are essentially noninfectious viral shells that mimic the structure of viruses but lack the viral genome.^[^ ^57^ ^]^ VLPs have been used to package nuclease mRNA or Cas9 protein and RNP complex into viral capsids through fusion or ABP/aptamer interaction strategies.^[^ ^57^, ^58^ ^]^ Reactive amino acids, including cysteine residues in these VLPs, allow for chemical modification of both their interior and exterior to engineer VLPs for specific delivery goals.^[^ ^59^ ^]^

Inorganic carrier particles, such as gold NPs, carbon nanotubes, mesoporous silica, and polymeric particles, have also emerged as promising tools for CRISPR delivery due to their unique physicochemical properties.^[^ ^32^, ^60^ ^]^ These particles can be engineered to carry CRISPR components and protect them from degradation, ensuring efficient delivery into target cells. The surface of inorganic particles can also be functionalized with ligands or antibodies to enhance specificity and facilitate targeted delivery for gene delivery in vivo. However, while ligand‐targeted delivery can improve selectivity, it is not clear whether this selectivity boost will be sufficient to minimize side effects in clinical applications. Finally, many inorganic and polymeric particles offer advantages such as controlled release and often minimal toxicity, making them suitable for both in vitro and in vivo studies. In this review, we discuss the latest findings on the use of viral and nonviral engineered particles for delivering CRISPR‐based genome editing therapeutics.

## 2 Overview of CRISPR/Cas Technology

In nature, CRISPR is a microbial adaptive immune system which incorporates DNA fragments from an invading virus or phage into the host genome as a memory mechanism that is used to degrade the invader's genome upon subsequent infection of the host.^[^ ^61^, ^62^ ^]^ Initially, Ishino encountered CRISPR sequences in *Escherichia* *coli* while isolating another gene,^[^ ^63^ ^]^ and CRISPRs were subsequently detected in many other microorganisms.^[^ ^64^, ^65^, ^66^, ^67^, ^68^ ^]^ The current classification of CRISPR systems includes 2 classes, 6 types, and 33 subtypes, and new systems are continuously discovered.^[^ ^69^ ^]^ Class 1 CRISPR systems make up 90% of known CRISPRs and feature an effector module composed of multiple Cas proteins that form a CRISPR‐associated complex for antiviral defense, include types I, III, and IV, and have also been adapted for use in genome engineering.^[^ ^70^, ^71^, ^72^ ^]^

Class 2 CRISPR systems have a single multidomain Cas effector protein that functions analogously to the class 1 complex, and includes type II, V, and VI.^[^ ^73^ ^]^ The type II CRISPR system is best understood for gene‐engineering applications due to its simplicity and the extensive studies conducted on the structure and function of the Cas9 protein.^[^ ^74^, ^75^ ^]^ More recently, a class 2 type VI RNA‐targeting subset of CRISPR systems was discovered, which has opened up many potential applications for direct RNA targeting as well as the use of CRISPR for molecular diagnostics based on properties of the first well‐characterized RNA‐editing Cas13a protein (formerly C2c2) which possesses a nonspecific collateral ribonuclease activity.^[^ ^76^ ^]^ This collateral RNase activity has been adapted for the detection of trace amounts of nucleic acids and has given rise to CRISPR‐based diagnostic platforms.^[^ ^77^, ^78^ ^]^ Finally, strategies used for the discovery of CRISPR systems are now applied for the mining of microbial genomes for other potential genome engineering proteins such as the large serine recombinases—enzymes that can catalyze the insertion of much longer DNA fragments for large‐scale genome editing.^[^ ^79^ ^]^

**Figure 2:**
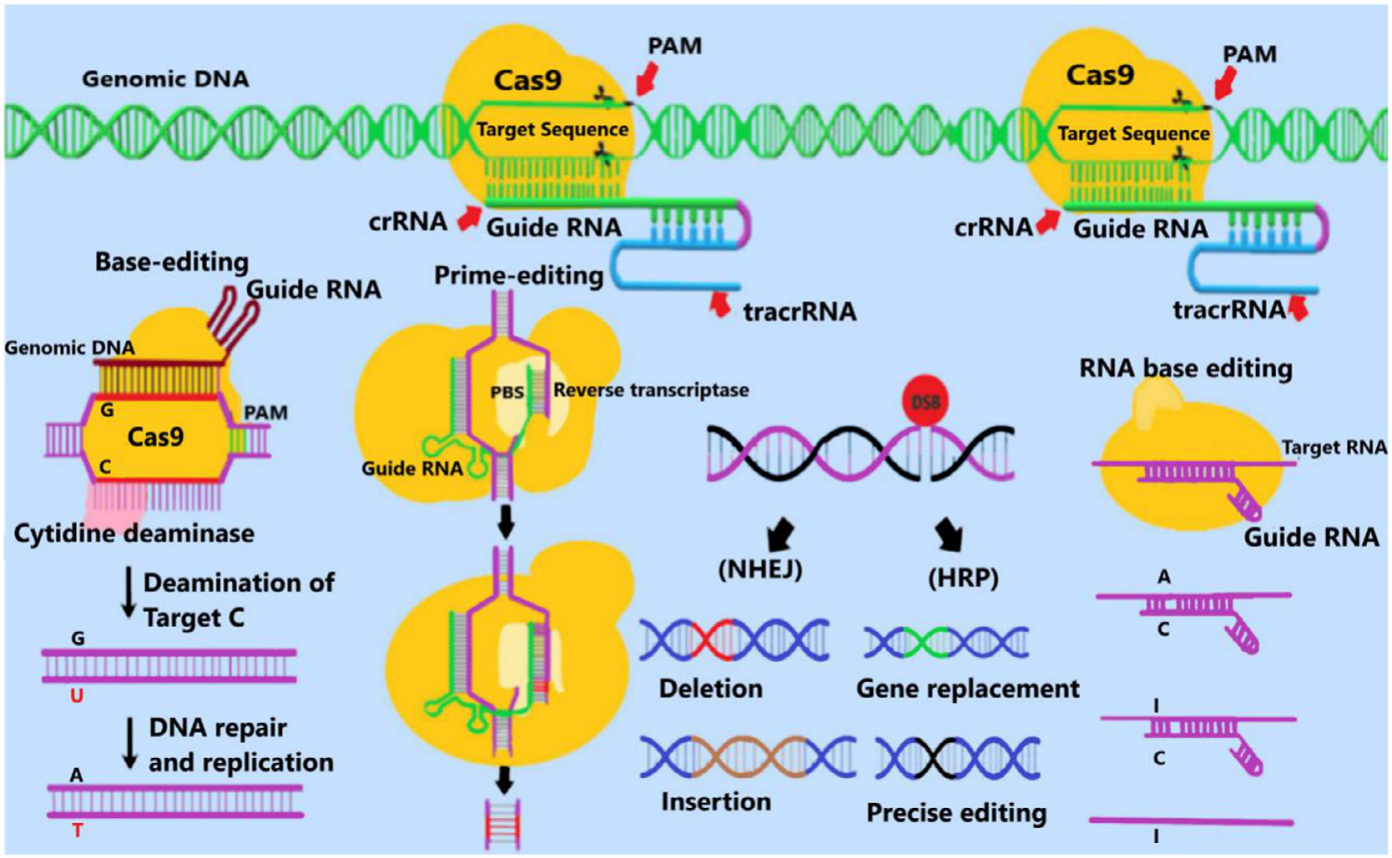
Schematic representation of the CRISPR function mechanism. The CRISPR system is used for genome editing in two manners including HDR and NHEJ. In HDR, DNA sequence homology is used for gene editing in the correct genomic location. In contrast, NHEJ works by identifying the broken ends of DNA and sticking them back together.

The Cas9 protein from *S. pyogenes* (SpCas9) has been used as the prototypical system to understand the mechanism by which CRISPR mediates genomic editing.^[^ ^80^ ^]^ In bacteria, Cas9 binds to a specific crRNA and a scaffolding trans‐activating CRISPR RNA (tracrRNA) which together are required for recruitment of the complex to target DNA sites and mediation of DSBs. In genome editing contexts, a shorter chimeric guide RNA molecule is engineered to replace the crRNA/tracrRNA hybrid.^[^ ^81^ ^]^ Within the crRNA, a 20 bp protospacer sequence is responsible for the sequence‐specificity of Cas9 targeting, these 20 bp match the genomic DNA target. Once bound to the target Cas9 unwinds the dsDNA and acts as a molecular pair of scissors to cut both strands. The Cas9 protein has six domains, REC I, REC II, Bridge Helix, protospacer adjacent motif (PAM) interacting, HNH, and RuvC.^[^ ^82^, ^83^ ^]^ The NH (His–Asn–His) nuclease domain cleaves the target DNA strand complementary to the gRNA, and the RuvC nuclease domain (including RuvC‐I, ‐II, and ‐III subdomains) cleaves the noncomplementary DNA strand to generate blunt ends.^[^ ^84^, ^85^, ^86^ ^]^ The PAM, comprising a few bases (5′‐NGG‐3′ for Cas9) downstream of the targeted DNA site, is required for Cas9's RNA‐guided targeting of genomic loci (**Figure** **2**). The PAM sequence plays an important role in determining the specificity and performance of genome editing, and varies for different CRISPR systems. Cas9 catalyzes CRISPR DNA cleavage 3 nucleotides upstream of the PAM site.

The DSB in the DNA induced by Cas9 cleavage is repaired by the endogenous cellular DNA damage repair machinery. The two major DNA repair pathways active in eukaryotic cells are 1) the high fidelity homologous directed repair (HDR) pathway which is restricted mostly to diving cells and requires homologous recombination; and 2) the error‐prone nonhomologous end joining (NHEJ) pathway which joins the ends of the DSB often resulting in an indel, and is the predominant repair pathway in somatic cells. It is the NHEJ or HDR mechanism repair of the Cas9‐induced DSB that leads to the editing at the target genomic site.^[^ ^87^, ^88^, ^89^ ^]^

Naturally occurring Cas protein orthologs, such as SaCas9, NmCas9, and Cas12a,^[^ ^90^, ^91^, ^92^ ^]^ offer options with varying protein sizes, which is beneficial when compactness is necessary. They also allow targeting of different genomic regions with alternative PAM sequences, provide various degrees of fidelity and specificity, and in the case of Cas12a, facilitate easy multiplexing. These factors are important considerations when designing a customized CRISPR therapeutic.

In addition to the natural diversity, the system is amenable to engineering to create Cas proteins with novel properties and CRISPR platforms with customizable functions. Mutating residues D10A and H841A in the nuclease domains of Cas9 results in the generation of a dead Cas9 (dCas9) protein, which retains the ability for site‐specific sequence binding without cutting the DNA.^[^ ^93^ ^]^ Instead, the dCas9 can be linked to another effector domain that can direct DNA modifications or modulate gene expression. Examples include transcriptional activation or repression, epigenetic modification of DNA by methylation or histone modification, and labeling of genomic regions.^[^ ^94^, ^95^ ^]^ Such a dCas9‐effector module system can have many potential clinical applications in the future. In sum, the combined natural diversity of Cas protein orthologs and the ability to edit and complex them with other protein effectors offers an unlimited array of applications of CRISPR platform‐based applications.^[^ ^96^ ^]^

Although much is understood about the CRISPR/Cas9 system and early clinical trials are based on this system, in the long term a DSB‐based genome editing approach is not optimal for human therapeutics as DSBs can be mutagenic and lead to genomic instability. Furthermore, to use Cas9 RNP for precise genome editing requires the simultaneous delivery of a DNA template and relies on HDR DNA repair pathways which have low activity in nondividing cells, yielding a low efficiency approach for somatic cell therapies. To overcome these challenges, a new generation of dCas9 systems were engineered for single‐base pair substitutions—Base Editing^[^ ^97^, ^98^, ^99^ ^]^ and longer sequence precise edits—Prime Editing,^[^ ^98^, ^100^ ^]^ without the need for a DNA template or reliance on HDR, and both can be reduced to protein and RNA components. Although still being perfected for efficiency and specificity, these CRISPR 2.0 and CRISPR 3.0 tools are likely to be the relevant approaches for future human therapeutics development.^[^ ^101^ ^]^

## 3 Challenges in NP‐Mediated CRISPR Delivery

NPs are materials whose particles range in size from 1 to 1000 nm. When used as drug carriers, NPs can be up to 100 nm in size in at least one dimension and are synthesized by using various materials such as synthetic or natural polymers, metals, or lipids. NPs are efficient delivery systems due to their ability to be more readily taken up by cells than larger molecules. Nanocarriers prevent drugs from disintegrating or being cleared too quickly, which leads to higher drug concentrations in the targeted tissues. Therapeutic molecules can either be situated on the surface of NPs or encapsulated within them. NPs can transport drug conjugates (consisting of both NPs and drugs) into specific cells using either passive or active transport mechanisms. Manipulating physical factors such as temperature and pH levels of the environment can also facilitate active transport. In contrast, passive transport utilizes the phenomenon of enhanced vascular permeability and the retention of both small and large molecules in tissues.^[^ ^102^ ^]^

**Figure 3:**
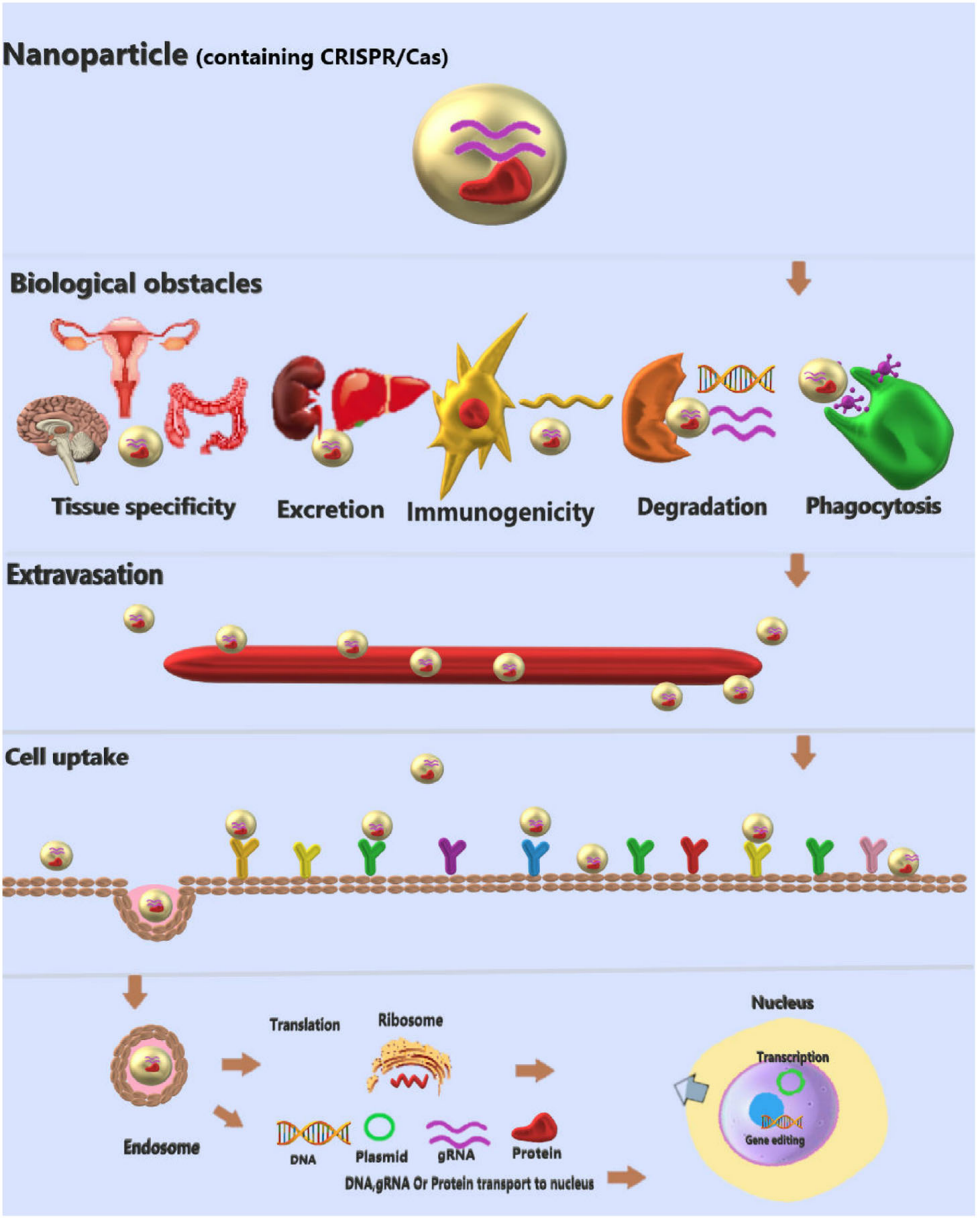
Schematic depicting the physical and biological obstacles encountered during the delivery of CRISPR components into cells. Engineered carriers facilitate the delivery of CRISPR to target tissues by overcoming these biological barriers.

Recently, nanocarriers such as lipids, gold, polymer, and DNA have been successfully employed in CRISPR therapies for delivering the CRISPR/Cas components to target cells. Several studies have shown that NPs can be an effective solution for the delivery of CRISPR/Cas components to cells, tissues, and organs.^[^ ^32^ ^]^ Several nonviral delivery strategies are used for therapeutic purposes but effective and safe gene editing remains a major challenge (**Figure** **3**). The delivery is primarily determined by the design and specificity of the guide RNA and the safe, efficient delivery vector is the major obstacle for NP‐mediated delivery of CRISPR. The physical delivery method appeared to be suitable for most in vitro applications, despite its high efficiency,^[^ ^49^ ^]^ but not for in vivo ones. Immunogenicity, carcinogenesis, limited DNA packaging capacity, and scale‐up production are common concerns for the viral vector approach as it transitions into clinical‐grade therapeutics.^[^ ^103^ ^]^ The size of the Cas protein is very large, so it is difficult to package it into a single vector. Therefore, supplementary gRNA vectors, either in DNA or RNA form, also must be delivered simultaneously.^[^ ^104^ ^]^ However, compact versions of Cas proteins have been developed, such as Cas9 nickase, which is about half the size of the full‐length Cas9 protein. This smaller size makes it easier to package into viral vectors, such as AAV vectors.^[^ ^50^ ^]^

After administration to cells, it is important for CRISPR components to withstand all the intracellular and extracellular barriers so that they can stably reach their target location. In order to successfully deliver CRISPR components, a nonviral delivery system must first overcome extracellular enzymes such as RNases, proteases, DNases, and macrophages present in the blood. These barriers could cause the degradation and phagocytosis of the nonviral delivery system comprising CRISPR components. Furthermore, the reticuloendothelial system present in the spleen and liver may result in the eradication of vectors. Therefore, chemical modifications such as PEGylation are required to protect the inorganic vectors as well as the CRISPR components from the host immune response and enzymatic degradation. The second hurdle is the delivery of the CRISPR construct and reduction of possible off‐target effects in undesirable regions.^[^ ^103^ ^]^

The NPs confront a variety of obstacles once they are in circulation. After being opsonized by blood proteins, they may then be identified by the mononuclear phagocyte system (MPS) cells and eliminated once in circulation. The NP population that is not cleared by the MPS can efficiently extravasate through the endothelial lining and into the surrounding tissues in order to exit the circulation.^[^ ^105^ ^]^ Therefore, to enhance the accumulation of NPs in the appropriate organ various mechanisms such as retention effect in solid tumors and improved permeability are employed.^[^ ^103^ ^]^ Permeability can be improved by using materials that can temporarily disrupt the tight junctions between endothelial cells that line the blood vessels. Another approach to improve NP permeability is by exploiting the characteristics of the target tissue. For instance, tumors often have leaky blood vessels and a deficient lymphatic system, which allows NPs to accumulate in the tumor tissue more easily than in normal tissue. This phenomenon is known as the enhanced permeability and retention effect, and it can be used to target NPs to solid tumors.^[^ ^106^ ^]^

Furthermore, active peptides or ligands, which bind and recognize cell‐type specific or tissue‐specific receptors or extracellular molecules, have been chemically attached to the surface of NPs. These ligands guide the cargo for accurately reaching a specific location, such as a specific cell or tissue, for targeted gene editing. Once they reach the targeted cells, the ligands are internalized, allowing for precise delivery of the gene‐editing cargo.^[^ ^107^, ^108^ ^]^ However, the delivery process is greatly influenced by the size of the NPs, i.e., small NPs are usually preferred vehicles for gene editing. Small NPs (<100 nm) have been shown to cross cell membranes more easily, allowing the intracellular and delivery of CRISPR constructs. As a result, the targeting efficiency is dramatically enhanced.^[^ ^109^ ^]^ One of the main limitations of small NPs is the reduced specificity, which may lead to off‐target effects. Conversely, small NPs have shown limitations to few bioresponsive stimulating factors and thus remain unused for clinical applications. So, the delivery carriers design with diverse biological stimuli is further needed for precise control of the CRISPR/Cas system.^[^ ^110^ ^]^

After the NPs reach the target cells, the final challenge is their intraendosomal degradation (at pH 5.0). To evade endosomal degradation, NPs can be modified by using endosomolytic agents such as polymers, proteins, peptides, toxins, and small chemical compounds.^[^ ^111^ ^]^ For successful genome editing, more advanced techniques are required to further shield the entire delivery process (**Table** **1**).^[^ ^103^ ^]^ To make gene editing a more robust tool, research has been focused on improving the accuracy and efficiency of the CRISPR gene‐editing technology in treating human diseases.

**Table 1:** Advantages and disadvantages of NPs for CRISPR delivery.

NPsa), b)	Advantages	Disadvantages	References
AuNPs	Easy surface modifications	High accumulation in the liver and spleen causes organ damage	[216, 363, 364]
	High cellular uptake		
	Targeted delivery		
	Minimal off‐target delivery		
	Easy to synthesize		
	High loading efficiency		
	Controlled dispersity		
LNPs	Easy modifications	Low delivery efficiency	[216]
	Economical		
	Low immunogenicity	Low stability	
	Easy preparation		
Polymeric NPs	High stability	Low efficiency	[216]
	Low immunogenicity	Insufficient toxicological reports	
	Affordable		
	High loading capacity		
	Tunable physicochemical properties		
Albumin‐based NPs	Trivial synthesis	Batch‐to‐batch variability	[216]
	Nontoxic		
	Nonimmunogenic		
	High loading capacity		
	Biocompatible		
	Biodegradable		
DNA‐based NPs	Controllable structure and size	Complex structure	[216, 365]
	Programmable	Poor stability	
	Nontoxic	Immunogenicity	
		Off‐target gene regulation	
		Pharmacokinetics and pharmacodynamics	
		Costly	

a) LNP, lipid‐based NP;

b) AuNPs, gold NPs.

## 4 Applications of AuNPs in CRISPR Delivery

Metal NPs accompanying CRISPR technology have several diagnostic and therapeutic applications. A major drawback of using metals as nanocarriers is their potential long‐term toxicity.^[^ ^112^, ^113^ ^]^ In contrast, beneficial physicochemical properties such as high reactivity, stability, and photothermal and plasmonic properties of metal NPs make them potent carriers for therapeutic agents.^[^ ^114^ ^]^ AuNPs are among the most widely used in the field of metal NPs (**Table** **2**). Due to good biocompatibility, chemical inertness, strong fluorescence emission, and large specific surface area, AuNPs have garnered special attention in the field of bioimaging and biosensing and can be used as a drug delivery platform.

**Table 2:** AuNPs used in genome editing.

NPa)–i)	Strategies for delivering CRISPR cargo	Target gene	Application	Disease	Properties	References
AuNP	Plasmid encoding Cas9 and gRNA	PLK1	AuNPs release into the cytoplasm through laser‐triggered thermoeffects and enter the cell nucleus with TAT guidance, effectively knocking out the PLK1 gene in melanoma tumors and inhibiting tumor growth in vitro and in vivo	Melanoma	A lipid/AuNPs complex, with the inner AuNPs core functioning not only as a plasmid carrier but also as a photothermal release agent, has the ability to carry large‐sized (≈10,000 bp) plasmids encoding Cas9/gRNA	[264]
AuNP	RNP complex and Cas9–gRNA plasmid	CXCR4, BFP, and dystrophin gene	Correction of the DNA mutation causing murine Duchenne muscular dystrophy (DMD) by local injection shows minimal off‐target DNA damage	DMD	A vehicle (CRISPR‐Gold) for gene editing via homology‐directed repair, enabling simultaneous in vivo delivery of Cas9 protein, guide RNA, and donor DNA, containing an endosomal disruptive polymer PAsp (DET)	[119]
AuNC	Cas9 protein and gRNA	E6	Knockdown of the E6 oncogene, eventually inducing p53‐dependent apoptosis in cervical cancer cells with minimal effect on normal human cells	Cervical cancer	A pH‐dependent self‐assembly of AuNCs with SpCas9 protein under physiological conditions. SpCas9‐AuNCs are stable at high pH but are disassembled at low pH	[121]
AuNC	Cas9 protein and gRNA	PLK1	Reduction of PLK1 protein expression by more than 70% in the A375 cell line inhibits melanoma cancer progression	Melanoma	A nanocarrier with a core of AuNC and a shell of lipids, facilitating electrostatic interactions between the core (TAT‐AuNCs), gene‐editing agents (Cas9 protein/sgPLK1 plasmid), and the shell (lipid)	[113]
Gold nanorod	gRNA/Cas9 complex	EGFP and PLK1	Gene editing combined with photothermal therapy	Breast cancer	A gold nanorod modified with TAT and Linker‐Aptamer to load the gRNA/Cas9 complex, which can also be exploited for mild photothermal therapy	[263]
Arginine Functionalized AuNP	Cas9 protein and gRNA	Human AAVS1 gene, human PTEN gene	Offers a promising nuclear and cytoplasmic delivery (≈90%) of Cas proteins, with genome editing effectiveness ranging from 23% to 30%	Cancer	Laboratory synthesis of AuNP can take up to a few weeks but can be synthesized in large batches that can be used for many years without compromising quality	[116]
AuNP	Cas12 protein and gRNA	Telomerase	Highly accurate telomerase activity assay for clinical cancer diagnosis	Liver cancer	Rapid (≤15 min) and reliable detection, offering a convenient and user‐friendly telomerase activity assay	[123]
AuNP	Cas12 protein and gRNA	E, N, O	More suitable for sensitive visual detection	SARS‐CoV‐2 detection	Detection is possible with the naked eye, with a detection limit of 50 RNA copies per reaction	[124]

a) AuNCs, gold NP clusters;

b) BFP, blue fluorescent protein;

c) CXCR4, C–X–C motif chemokine receptor 4;

d) E, small envelope protein gene;

e) EGFP, enhanced green fluorescent protein;

f) N, nucleocapsid protein gene;

g) O, open reading frame 1ab;

h) SMOF, silica–metal–organic framework hybrid NP;

i) PLK1, polo‐like kinase 1.

AuNP‐mediated delivery of CRISPR is considered to be one of the most effective and promising strategies in treating various diseases such as cancer. AuNPs are considered an emerging class of vectors for RNP–CRISPR‐mediated genome editing. The hydrophobic nature and surface charge of AuNPs can be easily adjusted by reaction with sulfhydryl (SH)‐containing compounds, forming Au—S bonds.^[^ ^115^ ^]^

A study conducted by Mout et al. prepared AuNPs bound with an arginine functional group.^[^ ^116^ ^]^ These NPs were used for the in vitro or in vivo delivery of gRNA and Cas9 protein. In their work, Cas9 was N‐terminally tagged with a glutamate peptide. As a result, the negatively charged peptide tags neutralized the positively charged Cas9 protein, generating self‐assembled nanostructures by binding to the arginine residues on ArgNPs. This approach offers a promising nuclear and cytoplasmic delivery (≈90%) of Cas proteins and genome editing effectiveness ranging from 23% to 30%.^[^ ^116^, ^117^, ^118^ ^]^

In the gene editing era, the AuNP applications have been propelled by the advancement of the CRISPR‐Gold technology. This technology can adjust the quantity per injection as well as decrease the side effects of CRISPR systems. CRISPR‐Gold comprises AuNPs conjugated to DNA‐Thiol, which are further complexed to donor DNA, Cas9 RNP, as well as a cationic endosomal disruptive polymer termed poly(*N*‐(*N*‐(2‐aminoethyl)‐2‐aminoethyl) aspartamide). The dystrophin gene can be repaired using CRISPR‐Gold technology with a 5.4% homology‐directed repair efficiency. Through this technique, muscle fibrosis can be minimized in X‐linked muscular dystrophy in mice.^[^ ^119^ ^]^

CRISPR‐Gold technology has been proposed for treating various neurological and social disorders because this tool can edit specific brain cells such as neurons, microglia, and astrocytes. Autism in the adult mouse caused by the fragile X syndrome can be reversed and the level of metabotropic glutamate receptor 5 (mGluR5) can be reduced by the transfer of the RNA‐guided nucleases Cas9 as well as Cpf1 via intracranial injection.^[^ ^120^ ^]^ CRISPR‐Gold technology can treat both single gene and polygene diseases. Due to the brilliant emission of fluorescence and surface functionalization, AuNCs can be utilized to monitor biological effects during the gene editing process by using confocal laser scanning microscopy.^[^ ^112^ ^]^ Exploring increased production of Au‐based nanocomposites can enhance the therapeutic and diagnostic applications of CRISPR technology. Also, self‐assembly of an AuNC with the Cas9 protein enables efficient delivery into the cell nucleus. This process, highly pH‐dependent, resulted in an efficient knockdown of the E6 oncogene. Eventually, this process induced p53‐dependent apoptosis in cervical cancer cells with minimal effect on normal human cells.^[^ ^121^ ^]^

Recently, CRISPR technologies have also gained attention for their potential applications because of their potential use in molecular diagnostics. The Cas12a protein possesses a unique collateral cleavage activity, allowing indiscriminate cleavage of ambient single‐stranded RNA after activation by matched DNA targets. Because of this property, the CRISPR/Cas12a system along with AuNPs has been actively employed in biosensing.^[^ ^122^ ^]^ A colorimetric code platform was developed based on programmable CRISPR/Cas12a technology and AuNPs probes to enhance the telomeric repeat amplification protocol method, enabling rapid (within 15 min) and reliable detection of telomerase activity. This platform was used to analyze clinical specimens of liver cancer, accurately detecting their telomerase activity with 93.75% sensitivity and specificity.^[^ ^123^ ^]^ In addition, a colorimetric method based on CRISPR/Cas12a and recombinase polymerase amplification assay was developed for SARS‐CoV‐2 detection, with a detection limit of 50 RNA copies per reaction. In this method, a magnetic pull‐down was used to capture AuNP probes, providing a more manageable approach to visually analyze the *trans*‐cleavage reaction. AuNP's molar absorption coefficient is multiple times larger than that of fluorescent dye molecules, which makes it more suitable for sensitive visual detection; therefore, CRISPR/Cas12a‐based detection relies mainly on the AuNP probe.^[^ ^124^ ^]^

In another study, to detect multidrug‐resistant (MDR) bacteria, a CRISPR/dCas9‐mediated surface‐enhanced Raman scattering assay was developed. In this method, dCas9/gRNA RNPs in combination with Au‐coated magnetic NPs were designed to recognize the genes of MDR bacteria and could detect MDR bacteria without any purification or gene amplification steps.^[^ ^125^ ^]^

AuNPs typically demonstrate excellent biocompatibility, implying they may be less toxic than certain other CRISPR delivery methods. However, ensuring their safety remains essential.^[^ ^37^, ^126^ ^]^ While they offer potential benefits, several important factors must be considered: 1) Size‐dependent effects: Research indicates that AuNPs ranging from 5 to 30 nm can induce cell death and oxidative stress in cell cultures.^[^ ^127^ ^]^ 2) Inconsistent findings: While in vitro studies highlight potential risks, research on tissues such as the retina indicates that these effects may not always occur in living organisms.^[^ ^128^ ^]^ 3) Long‐term impact: Most research to date focuses on short‐term effects, leaving the long‐term impacts of AuNPs in the body uncertain.^[^ ^129^ ^]^ and 4) Excretion and toxicity: The processes of how AuNPs are removed from the body and their possible long‐term toxicity are still being studied.^[^ ^130^ ^]^

The safety profile of AuNPs in CRISPR delivery is still under investigation. Further research is needed to fully comprehend their long‐term effects and create strategies to ensure their safe application in gene therapy. Scientists are actively working on creating safer and more efficient CRISPR delivery systems using AuNPs. Some promising methods include stimuli‐responsive designs, where these AuNPs react to specific triggers to enable controlled release of CRISPR components, potentially reducing unintended side effects, and hybrid materials, which involve integrating AuNPs with other materials, such as polymers, to enhance safety and functionality.^[^ ^131^ ^]^

## 5 Polymeric NPs: Promising Carriers for CRISPR Delivery

Nonviral delivery systems such as polymeric NPs offer several advantages, including quick breakdown, minimal immunogenicity, low cost, tunable physicochemical properties (such as the ability to modify weight, shape, size, and charge), high loading capacity, affordability, and high stability (Table 1).^[^ ^132^, ^133^, ^134^, ^135^ ^]^ Polymeric NPs can be synthesized from natural polymers (such as chitosan and sodium alginate), semisynthetic, or synthetic polymers (such as poly lactic‐*co*‐glycolic acid (PLGA) and polyacrylamide) and may take on various shapes and forms, including dendrimers, hydrogels,^[^ ^136^, ^137^, ^138^, ^139^, ^140^, ^141^, ^142^, ^143^, ^144^, ^145^, ^146^, ^147^, ^148^, ^149^, ^150^, ^151^, ^152^, ^153^, ^154^, ^155^ ^]^ and nanomicelles.^[^ ^156^, ^157^ ^]^ They play essential roles in various applications, including the delivery of drugs, medical imaging, and the diagnosis of diseases.^[^ ^157^ ^]^

The rapid development of nanotechnology has rendered nanocarriers potent nonviral delivery methods for the CRISPR system.^[^ ^158^, ^159^ ^]^ The use of polymeric carriers to deliver CRISPR genome editing components has shown high success rates in genome editing.^[^ ^160^ ^]^ Among the polymers, poly β‐amino ester (PBAE) is a widely utilized cationic polymeric carriers for genomes with superior biocompatibility, biodegradability, accessibility, and affordability.^[^ ^161^ ^]^ CRISPR constituent delivery by employing tailored polymeric NPs has the ability to improve the safety and efficacy of the genome editing process. Recent research has used polymeric NPs to deliver CRISPR gene‐editing components in several different ways (**Table** **3**).^[^ ^158^, ^160^, ^162^, ^163^, ^164^ ^]^ The PBAE is a type of biodegradable cationic polymer that is useful in delivering plasmid DNA.^[^ ^165^ ^]^ Also recently, the ability to use polymeric NPs (including a different PBAE formulation) to deliver CRISPR gene‐editing components in the form of plasmid DNA has been developed.^[^ ^166^ ^]^

**Table 3:** Polymeric NPs used in genome editing for CRISPR delivery.

Nanopolymera)–j)	Strategies for delivering CRISPR cargo	Target gene	Application	Disease	Properties	References
PBAE	Plasmid encoding Cas9 and gRNA	GFP and E7	Provide new insights into screening/transfection requirements for constructing nonviral CRISPR delivery systems	Cervical cancer	Biocompatibility, biodegradability, ease of acquisition, and low cost	[161, 366]
PLGA	Plasmid encoding Cas9 and gRNA	Cdk5	Suppress PD‐L1 expression on malignant cells	Colorectal cancer immunotherapy	Quick breakdown, low cost, high loading capacity, affordability, and high stability	[163]
PLGA	Encapsulating Cas9 protein and single gRNA	γ‐globin	In vivo therapy of hemoglobinopathies and other genetic diseases	Hemoglobinopathies	Biodegradability, compatibility with production methods, and a stable linker to PEG	[158]
PBA	Polymer NP carrying Cas9 mRNA and gRNA	p53	Knock out the gene expression of cancer cells (HeLa)	Cervical cancer	PBA can recognize sialic acid (SA) and can bind it to generate a stable borate ester, both at healthy pH levels and in acidic environments like tumors	[160, 367]
PEG	RNP complex and plasmid encoding Cas9, Cas12a and gRNA	PDS and IPK	Knock out gene expression in maize protoplasts and bananas	Phytic acid accumulation (a type of Plants disease)	Requires less time, providing a quick and efficient method for plant gRNA validation and transient expression tests	[170, 171]
Positively charged chitosan	RNP complex	RFP and CDK11	Decrease (>90%) in CDK11 protein	Breast cancer	Inexpensive, biocompatible, noncytotoxic, and biodegradable	[179, 180]

a) Cdk5, cyclin‐dependent kinase 5;

b) CDK11, cyclin‐dependent kinase 11;

c) HBB, hemoglobin subunit beta;

d) IPK, inositol phosphate kinase;

e) PBA, phenylboronic acid;

f) PDS, phytoene desaturase;

g) PEG, polyethylene glycol;

h) PEI, polyethyleneimine;

i) RFP, red fluorescent protein;

j) RHBDF1, rhomboid 5 homolog 1.

PLGA is a functional biodegradable polymer that is typically produced by the ring‐opening copolymerization of lactide and glycolide. Because of its biocompatibility, biodegradability, and excellent safety profile, PLGA has been approved by FDA as a particularly beneficial and widely used carrier for drug delivery.^[^ ^167^ ^]^ Many groups have employed PLGA‐based NPs to deliver CRISPR and demonstrated effective editing via plasmid DNA encoding the Cas9 endonuclease and gRNA into different cell lines.^[^ ^161^, ^168^, ^169^ ^]^ CRISPR–PLGA NPs encapsulating Cas9 protein and gRNA were delivered specifically to HUDEP‐2 cells, primary erythroblasts, and CD34^+^ cells,^[^ ^158^ ^]^ and to target Cdk5 to suppress programmed death ligand‐1 (PD‐L1) expression on malignant cells as a genetically operated checkpoint inhibitor therapy.^[^ ^163^ ^]^

PEGylated NPs are another frequently utilized nanocarrier due to their ease of operation, high transfection efficiency, minimal equipment requirements, and ability to provide consistent results. Recently, the use of the PEGylated NPs as CRISPR cargo has been considered, and experiments have demonstrated that the polyethylene glycol (PEG)‐delivered CRISPR RNPs technology is suitable for gene editing.^[^ ^170^, ^171^ ^]^

Cationic polymeric NPs (CPNPs) exhibit greater chemical diversity as well as surface functionalization. They also offer greater options for designing flexible structural configurations. CPNPs have found widespread application in delivering various nucleic acids, including mRNA, plasmid DNA, and DNA (including oligo DNA, ssDNA, and dsDNA).^[^ ^172^, ^173^ ^]^ Among various cationic polymers, chitosan and polyethyleneimine (PEI) are considered the most popular vehicles for transporting CRISPR. Through an endocytic pathway, they penetrate the cell membrane through an endocytic pathway, shielding the loaded nucleic acid from nuclease degradation and immune responses. PEI, in particular, has shown significant transfection efficiency and is highly effective for gene delivery. PEI‐based systemic gene delivery surpasses other nonviral vectors such as DOTAP‐based liposomes, as PEI‐based polyplexes enter cells via a clathrin‐ and caveolae‐dependent pathway, which bypasses the lysosome and protects DNA from lysosomal enzymes.^[^ ^174^, ^175^, ^176^ ^]^ Furthermore, PEI's endosmotic action increases transfection efficiency over polycations like poly‐lysine. Studies have demonstrated that PEI can deliver Cas9/gRNA in vitro with efficiency equivalent to that of commercial lipofection reagents.^[^ ^177^, ^178^ ^]^

Qiao et al. have synthesized positively charged chitosan NPs loaded with a RFP for genome editing.^[^ ^179^ ^]^ The basic purpose of RFP‐chitosan‐based NPs was to deliver Cas9 RNP and 20 glutamate residues. To repair the genome, the single‐stranded DNA donor in this system was first carried to the cytoplasm where it would be liberated and then transported to the nucleus. Similarly, Liu et al. synthesized dual‐targeted cationic polymer hybrid NPs to successfully knock out the CDK11 gene in tumor cells. To successfully knock out the CDK11 gene in tumor cells, these dual‐targeted polymeric NPs directed the plasmid encoding CRISPR toward the nucleus, resulting in a therapeutic effect.^[^ ^180^ ^]^

Many researchers have synthesized multistage delivery NPs (MDNPs), including for tumor suppression. These nanostructures have been used for targeted administration, as a strategy for preventing tumor growth. The main benefit of these polymeric nanocarriers is their resilience to the acidic microenvironment of tumors.^[^ ^181^ ^]^ Chitosan is a component used to fabricate MDNPs for site‐targeted delivery of the CRISPR system. It possesses high mucoadhesion and penetration capabilities, making it an excellent choice for medication administration in mucosal and ocular regions. However, MDNPs are limited by several disadvantages, including insufficient toxicological data, toxicity (polyvinyl alcohol is widely used as a detergent, although it is somewhat toxic), low efficiency, and difficulties in synthesis.^[^ ^182^, ^183^, ^184^ ^]^ Thus, the clinical and translational future of MDNPs depends on the polymer chemistry community developing synthetically simple and low‐cost, nontoxic polymers, and the biomedical community rapidly testing such agents under physiological conditions.

Polymeric NPs hold great promise for drug and CRISPR delivery, as well as various biomedical applications. However, their interactions with the immune system and potential cytotoxic effects remain significant concerns in the body.^[^ ^185^ ^]^ Immune responses associated with polymeric NPs include: 1) Foreign object recognition: The immune system often identifies polymeric NPs as foreign objects, triggering an immune response. This recognition can lead to macrophages and other immune cells engulfing the NPs, potentially causing inflammation and subsequent removal from the body. 2) Granuloma formation: Upon entering the bloodstream, polymeric NPs can acquire a protein corona formed by blood plasma proteins. This corona alters interactions between the NPs and immune cells, influencing immune responses. 3) Dose‐dependent effects: The severity of immune responses typically increases with the administered dose of polymeric NPs. Higher doses may overwhelm the immune system, leading to more pronounced reactions such as heightened inflammation or immune rejection.^[^ ^185^, ^186^ ^]^ Certain cytotoxic effects such as oxidative stress and off‐target effects based on material properties may occur in vivo. Studies involving polymeric NPs are crucial for understanding their potential safety issues. These studies typically involve histological analysis, blood chemistry analysis, and organ function tests.^[^ ^35^, ^187^, ^188^ ^]^

Future research efforts should focus on developing more sophisticated in vivo models, identifying specific polymeric characteristics that minimize immune responses and cytotoxicity, and optimizing the design of polymeric NPs for targeted delivery and reduced off‐target effects.

## 6 Lipid NPs: Pioneering Advances in CRISPR Delivery

Lipid nanocarriers have emerged across the pharmaceutical industry as versatile nanomedicine delivery platforms. Their success is attributable to the relative ease of production and scale‐up, reduced immune responses, multidose capability, extensive and stable drug loading, and design flexibility. In a highly versatile platform, LNPs have been employed in drug delivery, including nucleic acid‐based drugs, and are currently in the spotlight as the lead clinical nonviral delivery system with extensive clinical trials and testing in humans.

Using LNPs as a delivery vehicle has other benefits, such as target‐specific delivery (by introducing targeting ligands on NP surfaces), endosomal escape (by destabilizing the endosomal membrane or enhancing fusion to the endosomal membrane through modifying the lipid compositions), high gene knockout efficiency, suitable drug release, and bypassing extracellular nuclease degradation. Positively charged lipids and negatively charged nucleic acids interact with one another through electrostatic and host–guest association to form a stable complex.^[^ ^189^, ^190^, ^191^ ^]^ Through endocytosis, this complex is taken up by cells. The ability of LNPs to carry both mRNA and siRNA has been widely verified through clinical studies. However, the delivery efficiency of LNPs carrying CRISPR components still needs to be improved. As a result, this approach does not yet satisfy the clinical standards for efficient genome editing. Improvement and modification of the LNP delivery system can significantly enhance its stability and delivery efficiency, which will pave the way for gene editing in treating clinical diseases in the near future.^[^ ^32^, ^192^ ^]^

The most well‐known version of LNPs, the liposome, was the earliest nanomedicine delivery platform approved and successfully employed in clinical applications. Liposomes can encapsulate hydrophilic drugs in their internal aqueous core and entrap hydrophobic drugs in the lipid bilayer's hydrocarbon chain region. Therefore, they can transport various molecules, such as small molecules, proteins, and nucleic acids. Cationic lipid–nucleic acid complexes, solid lipid NPs, and nanostructured lipid carriers comprise many of the next generations of LNPs whose structures have provided more complex architectures with enhanced stability and capabilities.^[^ ^193^ ^]^

Currently, cationic lipid–nucleic acid complexes, as a vital component of the COVID‐19 mRNA vaccine, have received intense global attention. The four essential components of this LNP system are ionizable cationic lipids, phospholipids, cholesterol, and PEG lipids. Cationic LNPs with unique properties can form a stable complex with anionic nucleic acids, protect them from nuclease degradation, and ultimately enable their efficient delivery to target cells. Therefore, they have become the most widely used nonviral delivery system for nucleic acid drugs.^[^ ^194^ ^]^ They have undergone extensive preclinical and clinical evaluation for the therapeutic application of CRISPR‐mediated genome editing.

A key obstacle to developing improved LNPs is the still‐limited understanding of their interactions with cells. To address this problem, a study using arrayed CRISPR screening was performed to identify critical modulatory mechanisms for functional LNP‐mRNA (MC3 lipid‐based LNP encapsulated mRNA) delivery. In that study, 44 genes that increase or inhibit LNP‐mRNA productive delivery were identified. Many of the genes are involved in key activities such as host cell transcription, protein ubiquitination, and intracellular trafficking.^[^ ^195^ ^]^

Overall LNPs are considered an appropriate and safe vehicle for delivering mRNA and CRISPR. Furthermore, LNPs are also used as a vector for the delivery of therapeutic genes. Guo et al. have created antibody‐conjugated nanolipogels that target tumors,^[^ ^196^ ^]^ and this approach has led to the efficient delivery of a CRISPR system to treat triple‐negative breast cancer in orthotopic MDA‐MB‐231 tumor‐bearing mice. This complex displayed an 81% gene knockout efficiency, leading to a tumor suppression rate of 77%. These findings suggest that LNPs are promising and have high potential to make CRISPR genome editing a novel precision medicine cancer therapeutic.

With the use of aptamer‐ or protein‐based targeting ligands, RNA‐carrying NPs have been directed in vivo to desired cells; nevertheless, systemic administration of the NPs to desired cells without the use of targeting ligands typically remains challenging. Interestingly, specific LNPs containing conformationally constrained lipids, so‐called constrained lipid NPs (cLNPs), were able to interact with T‐cells without the use of targeting ligands. Exploiting endogenous lipid trafficking is a substitute for, or indeed another route toward, “active” targeting; notably, the only RNA NP therapy approved by the FDA uses LNPs lacking ligands that are transported to hepatocytes by endogenous cholesterol transport. In the aforementioned study, the transport of NPs to T‐cells was promoted by exploiting natural trafficking mechanisms. cLNPs can deliver siRNA and gRNA to T‐cells at relatively low doses and, unlike previously reported LNPs, do not preferentially target hepatocytes. Despite observing protein silencing in T‐cells at 0.5 mg kg^−1^, this amount would need to be reduced by more than 30‐fold to be as potent as an FDA‐approved siRNA delivery vehicle in mice. These data indicate that lipids’ conformational state can alter LNP tropism,^[^ ^197^ ^]^ and form the foundation of a roadmap toward future nanomaterials that can specifically target immune cell types without ligands. It is likely that there are specific conformations of lipids and polymers that can reproducibly form on the surface of nanomaterials that target surface proteins/receptors on cell subsets that enable this selectivity. The selectivity could also arise due to the protein corona which forms on the surface of nanomaterials in the presence of serum proteins, which might also endow selectivity due to the specific proteins attracted and adsorbed to the surface. Future studies on these phenomena will need to clearly identify the mechanisms of selectivity of these nanomaterials. From mechanistic understanding, intelligently designed high‐throughput searches of NPs can reveal novel ligand‐free nanomaterials with outstanding tropism to cell subsets in vivo. With such tools, in vivo CRISPR delivery will be increasingly selectively targeted to cells, and therefore increasingly efficient and efficacious. Nevertheless, it has been challenging to control the uniformity, stability, and size of LNPs. Therefore, in vivo delivery of LNPs is limited to targeting brain, muscle, and inner ear diseases and is currently under continuing investigation.^[^ ^198^ ^]^

Research on developing rapid identification systems for LNP‐mediated RNP delivery with novel tropisms in vivo is ongoing. Lee et al. constructed a combinatorial library of bioreducible LNPs for the intracellular delivery of Cas9/gRNA. The best‐performing LNP candidates with high targeted gene knockout efficacy and relatively low cytotoxicity were pinpointed through in vitro screening and were then administered systemically to Balb/c mice for an in vivo biodistribution investigation using fluorescent dye‐labeled and RNP‐complexed LNPs.^[^ ^199^ ^]^ A system for systemically administered RNA delivery for gene editing in nonhepatic tissues (endothelial cells) was designed, known as titled Fast Identification of Nanoparticle Delivery (FIND). This system is capable of quantifying the cytosolic delivery of more than 100 LNPs carrying mRNA to any combination of cell types in vivo. FIND generated multiplexed readouts of functional mRNA delivery by combining the Cre‐Lox system and rationally designed DNA barcodes. They formulated the Cre mRNA and a unique DNA barcode into LNPs using high‐throughput microfluidics. This approach quantifies functional cytosolic drug delivery (where the drug is active) and distinguishes it from in vivo screening that quantifies biodistribution (where the drug localizes over time). Using FIND, over 250 LNPs were able to deliver mRNA to a number of cell types in vivo and two LNPs were identified (7C2 and 7C3) to deliver siRNA, gRNA, and mRNA efficiently to endothelial cells. These data showed that the FIND system could detect NPs with new tropisms in vivo.^[^ ^200^ ^]^ In addition, this research group, using the same approach, identified an LNP, bm1, capable of in vivo gRNA delivery to bone marrow endothelial cells (BMECs). Interestingly, the chemical analysis demonstrated that the BMEC tropism did not correlate with LNP size but rather with the structure of PEG and the presence of cholesterol.^[^ ^201^ ^]^ As discussed above, in vivo screening is likely to lead to the discovery of a complex relationship between NP surface, structure, and tropism, thereby informing researchers how simple, potentially small, chemical changes can control NP targeting.

Miller et al. reported the first successful LNP system for in vivo and in vitro codelivery of Cas9 mRNA and gRNA (sgLoxP), which were able to reduce target protein expression by more than 90%.^[^ ^202^ ^]^ In a recent study, an LNP‐mediated delivery system was developed that significantly edited the transthyretin gene in mouse liver for at least 12 months and reduced its serum protein levels by more than 97% with a single dose. The system, called LNP‐INT01, which coformulated Cas9 mRNA and gRNA into a single particle, is comprised of biodegradable and ionizable lipids termed LP01, helper lipids, and PEG‐DMG.^[^ ^203^ ^]^

In vivo gene editing therapeutics based on the Cas9 protein have practical limitations due to their instability and low efficiency of protein cargo delivery. Wang et al. reported that combining cationic bioreducible NPs with anionic Cas9/gRNA created an electrostatic assembly that could lead to efficient protein delivery and gene editing. For stable nanocomplex formation for protein delivery, electrostatic self‐assembly between lipid and protein was necessary. In this study, 12 bioreducible LNPs were synthesized, all of which were formulated using cholesterol, DOPE, and C16‐PEG2000‐ceramide. These lipids were suitable for the in vitro and in vivo delivery of functional proteins. When cultured in human cells, negatively supercharged Cre protein and Cas9:gRNA that were complexed with bioreducible lipids efficiently (more than 70%) engendered gene recombination and genome editing.^[^ ^204^, ^205^ ^]^ In several other studies, unique LNPs, including chalcogen‐containing lipid,^[^ ^206^ ^]^ noncationic LNP,^[^ ^207^ ^]^ lecithin nanoliposomal particle,^[^ ^208^ ^]^ and ionizable cationic lipids,^[^ ^198^ ^]^ have been designed for effective genome‐editing protein delivery (**Table** **4**).

**Table 4:** Lipid‐based nanocarriers for CRISPR delivery.

LNPa)–d)	Strategies for delivering CRISPR cargo	Target gene	Application	Disease	Properties	References
ZALs	Cas9 mRNA and gRNA	LoxP	Successful LNP system for in vivo and in vitro codelivery of long RNAs	Liver, kidney, and lung diseases	Simplifying CRISPR/Cas engineering with this approach	[202]
LNP‐INT01	Co‐formulate Cas9 mRNA and guide RNA into a single particle	Ttr	A single dose significantly edited the transthyretin gene in the mouse liver for at least 12 months and reduced its serum protein levels by >97%	Liver‐based genetic diseases	A biodegradable ionizable lipid, high levels of durable in vivo CRISPR‐mediated gene editing, ability to re‐administer	[203]
Aminoionizable lipid	Cas9 mRNA and gRNA	PLK1	A single intracerebral injection of CRISPR‐LNPs targeting PLK1 into invasive orthotopic glioblastoma	Brain tumors	A novel class of ionizable amino lipids based on hydrazine, hydroxylamine, and ethanolamine linkers are safe and nonimmunogenic after systemic administration	[209]
Cationic nanoliposome	Cas9/gRNA plasmid	HPV16 E6/E7	Intratumoral injections in nude mice significantly inhibited tumor growth without significant toxicity	HPV‐positive cervical cancer	A long‐circulating pH‐sensitive complex, stable in physiological conditions, but disassembles in cancerous tissues due to the slightly more acidic environment	[212]
Antibody‐conjugated nanolipogel	Cas9/gRNA plasmid	Lipocalin2	Displayed an 81% gene knockout efficiency, resulting in a tumor suppression rate of 77%	Triple‐negative breast cancer	A noncationic, deformable nanolipogel that is relatively safe for use in vivo effectively avoids endosome entrapment	[196]

a) HPV, human papillomavirus;

b) LoxP, locus of crossover in P1;

c) Ttr, transthyretin;

d) ZALs, zwitterionic amino lipids.

Low editing efficiency in tumors as a result of utilizing current delivery systems and the potential toxicity of existing delivery systems hamper the utilization of CRISPR technology in cancer treatment; therefore, improvements are required to ensure the safe and efficient delivery of CRISPR to tumors. Rosenblum et al. reported a safe, efficient LNP‐based system using a novel aminoionizable lipid to codeliver Cas9 mRNA and gRNA. An intracerebral injection of CRISPR‐LNPs against PLK1 into an invasive orthotopic glioblastoma model resulted in in vivo gene editing of 70%. This intervention subsequently inhibited tumor growth by 50% and improved survival by up to 30%.^[^ ^209^ ^]^

While cationic liposomes have the advantages of high cellular uptake and efficient escape from endosomes, their cationic charges could cause poor tumor penetration, nonspecific accumulation, and a short half‐life in blood circulation. In contrast, neutral liposomes display deeper penetration into tissues, with a sacrifice in cellular uptake. An example of this is neutral liposomes comprising lecithin, cholesterol, and 1,2‐dioleoyl‐*sn*‐glycerol‐3‐((*N*‐(5‐amino‐1‐carboxylpentyl) iminodiacetic acid)succinyl)‐(nickel salt) (DOGS‐NTA‐Ni), used to deliver the recombinant Cas9 protein and gRNA complex (against dipeptidyl peptidase‐4 gene) in a type 2 diabetes mellitus mice model. This nanocarrier was dye‐labeled, and NIR imaging was used to monitor the particle in vivo biodistribution over time. As expected, this lecithin‐based liposomal nanocarrier particle was observed to accumulate in the liver from 2 hours to a day following injection. According to the findings, the extended retention of this NP in the liver might be responsible for the improved efficacy of the gene disruption effect.^[^ ^208^, ^210^ ^]^ The NTLA‐2001 drug, developed by Intellia Therapeutics Company, is currently in phase 1 clinical trial (NCT04601051, Recruiting) in participants with hereditary transthyretin amyloidosis with polyneuropathy (ATTRv‐PN) to evaluate its safety, tolerability, pharmacokinetics, and pharmacodynamics. This drug comprises a lipid NP encapsulating CRISPR gene‐editing system (gRNA targeting transthyretin/Cas9 mRNA) and is administered intravenously.^[^ ^194^, ^211^ ^]^

Recent studies have focused on smart liposomes, those that undergo structural changes when exposed to microenvironmental stimuli including changes in temperature, pH, and the presence of a specific enzyme. With this “smart” strategy, selective homing and control of cargo release can be achieved at the target site. For example, a long‐circulating pH‐sensitive cationic nanoliposome complex displayed both excellent cell targeting and gene knockout rate (≈72%). These pH‐sensitive liposomes, stable under physiological conditions, disassemble in cancerous tissues due to the slightly acidic environment. The data showed that intratumoral injection of nanoliposome‐CRISPR gRNA‐HPV16 E6/E7 complex in nude mice with HPV‐positive cervical tumors significantly inhibited tumor growth without toxicity.^[^ ^212^ ^]^

Liu et al. developed a lipid compound called BAMEA‐O16B that is responsive to glutathione. This lipid is used to deliver Cas9 mRNA and sgRNA. The BAMEA‐O16B compound, which has hydrophobic tails containing disulfide bonds, can encapsulate RNA via electrostatic interactions. In the intracellular environment, which is glutathione rich, RNAs are efficiently released. The effectiveness of knocking down GFP expression in human embryonic kidney cells may be as high as 90% when Cas9 mRNA and sgRNA are delivered simultaneously using BAMEA‐O16B. Moreover, the BAMEA‐O16B/Cas9 mRNA/sgRNA NPs efficiently accumulate in hepatocytes and reduce the levels of proprotein convertase subtilisin/kexin type 9 in mouse serum to 20% of the nontreatment following intravenous administration.^[^ ^213^ ^]^

In a separate investigation, researchers designed a microbubble‐nanoliposomal (MB‐NL) particle to serve as a carrier for the Cas9/sgRNA RNP complex. This carrier allowed for targeted local delivery when activated by ultrasound. The protein constructs were successfully delivered into dermal papilla cells within the hair follicles of androgenic alopecia animal models through microbubble cavitation‐induced sonoporation. The targeted gene, SRD5A2, responsible for converting testosterone into a more potent androgen that contributes to hair loss, was effectively edited. This gene editing resulted in the suppression of SRD5A2 protein production, thereby promoting hair growth in the animal models. The results revealed that US‐activated MB‐NL (Cas9/sgRNA) treatment demonstrated significant gene editing efficiency (71.6%), with SRD5A2 mRNA levels showing an ≈70% reduction compared to the control. Additionally, there was a significant decrease in SRD5A2 protein expression, leading to a twofold increase in VEGF levels, which enhanced nutrient supply to the hair follicles and stimulated hair growth.^[^ ^214^ ^]^

Despite multiple ideas for spatiotemporal control of CRISPR/Cas9 gene editing, there has been no evidence of clinical applications for achieving precise regulation in a programmable and inducible manner while simultaneously minimizing off‐target effects. Currently, research on smart stimuli‐responsive CRISPR‐Cas9 delivery systems remains largely confined to preclinical evaluations. Therefore, concerted efforts are needed to advance these technologies into clinical applications.^[^ ^36^, ^131^ ^]^

## 7 Albumin‐Based NPs for CRISPR Delivery

Unlike other proteins, albumin possesses remarkable features such as high biocompatibility, high solubility, and low immunogenicity. Additionally, albumin is highly water soluble because of its overall negatively charged surface. There are several ligand‐binding regions, including Sudlow's site I (indole‐benzodiazepine site), on its surface which has the affinity to make bonds with dicarboxylic acids and large heterocyclic compounds. Moreover, Sudlow's site II (sudol site) tends to bind with aromatic carboxylic acids. Albumin is deemed very stable because of the presence of disulfide bonds which are formed internally by 34 cysteine residues. Furthermore, it possesses one free cysteine residue on the outer surface, which is important for ligand conjugation.^[^ ^215^ ^]^ These features make albumin‐based nanocarriers a suitable vehicle for delivering gene constructs and drugs. Albumin‐based NPs absorb charged molecules due to their high amino acid content.^[^ ^216^ ^]^ Under in vitro conditions, Cheng et al. successfully synthesized albumin‐based NPs displaying PD‐L1 and Cas9 protein.^[^ ^217^ ^]^ They showed that this complex successfully disrupts the target gene in colon carcinoma CT26 cells.

## 8 CRISPR Delivery via DNA‐Based NPs

Nadrian C. Seeman introduced DNA NPs which have been shown to exhibit promising therapeutic activity. It has become clear that DNA nanostructures display a broad range of applications, such as targeted drug delivery, genome editing, bioimaging, cancer therapy, and inflammation inhibition. The sequence of the DNA can be easily controlled, resulting in the self‐assembly of DNA into sophisticated nanostructures. DNA NPs are a novel class of NPs and they exhibit various applications such as diagnostics, environmental and biomedical.^[^ ^218^, ^219^ ^]^ Based on their numerous advantages such as biodegradability, biocompatibility, and strong loading capability, they are considered promising delivery vehicles.^[^ ^220^, ^221^ ^]^

Previously, the Watson–Crick DNA base pairing model was used to design DNA nanostructures. The disadvantage of this approach is that it requires large amounts of DNA for generating nanostructures. Advances in the rolling circle amplification (RCA) technique have made it easier for DNA to be assembled into nanostructures.^[^ ^222^ ^]^ Compared to older methods (such as DNA branched junctions) for designing DNA nanostructures, RCA requires small amounts of DNA. As a result, this method has significantly simplified the process of creating nanostructures.^[^ ^223^, ^224^ ^]^ For in vivo and in vitro RNP delivery, scientists have synthesized self‐assembled yarn‐like DNA nanostructures through this technique.^[^ ^225^ ^]^ To enhance their stability and cellular uptake, chemical modifications of DNA nanostructures were conducted. To facilitate gene delivery, DNA nanostructures have been modified with PEI.^[^ ^226^ ^]^ Furthermore, this strategy could potentially minimize off‐target delivery by allowing for more specific targeting of cells and ultimately, adverse side effects. Another study showed that modified NPs containing DNA/Cas9/gRNA exhibited better activity, resulting in 28% genome editing, along with increased endosomal escape and cellular uptake.^[^ ^225^ ^]^ DNA nanostructures have the ability to preferentially target certain tumor cells because ligands are coupled with the surface of the nanomaterials.^[^ ^227^, ^228^ ^]^ Researchers have shown that the utilization of stimulus‐responsive CRISPR/Cas9 gene editing tool engineered to activate or deactivate in response to specific environmental or cellular signals, enabling precise spatiotemporal control,^[^ ^110^ ^]^ in combination with a nanoflower can considerably increase the efficiency of genome editing.^[^ ^32^, ^229^ ^]^ Nanoflowers, flower‐shaped nanomaterials, are exciting due to their unique morphologies, straightforward synthetic routes, physicochemical properties, site‐specific action, potential for imaging, and controlled delivery of drugs.^[^ ^230^, ^231^ ^]^ In tumor cells, a nanostructures‐based delivery pathway called Cas9‐NF has been used to cross‐link Cas9 and polymeric micelles for the efficient intracellular delivery of Cas9. This strategy minimized the impact of the microenvironment on tumor cells. As a result, in a mouse tumor model, the expression of oncogenes was suppressed, resulting in a slow tumor growth.^[^ ^232^ ^]^ The downsides of DNA nanostructures include their liability but also sensitivity to temperature, ion strength, and nucleases. In addition, they are made of building blocks that are both pliable and extremely small, and as a result, it is difficult to identify or address the structure of individual nanostructures.^[^ ^233^ ^]^ Moreover, DNA nanostructures, like many foreign substances, could elicit an unfavorable immunological reaction. Chemical alteration of the bases and backbone of the DNA might help to alleviate this problem. Also, certain sequences which nonspecifically regulate gene expression in DNA strands may interact with messenger RNAs, miRNAs, or bind to DNA regulatory regions to influence gene expression. Furthermore, uncertainty exists about the pharmacokinetic and pharmacodynamic characteristics of DNA nanostructures and further research is necessary for their in vivo uses. The high cost of DNA nanostructures is another constraint as compared to substitutes like polymers which tend to be more affordable.^[^ ^234^ ^]^

## 9 Optimizing CRISPR Delivery with Aptamers

Aptamers, a type of single‐stranded nucleic acid, can be used as targeting ligands due to their specific biological affinities. Because of their novel features, such as selectivity, affinity, low cost, great specificity, and easy synthesis, they have gained remarkable importance and are used for genome editing and drug delivery. Many studies have reported that nanosized aptamers were packed along with the delivery cargo to increase their delivery efficiency.^[^ ^235^, ^236^ ^]^ Liu et al. used MCF‐7 cells to study breast cancer,^[^ ^237^ ^]^ under in vitro conditions, wherein chitosan‐based, self‐assembled aptamer‐targeted NPs were designed from endosomolytic peptide and AS_1411_ aptamer and this complex was employed to deliver sgCDK11‐Cas9 construct into tumor cells. According to their results, functionalized NPs with aptamer demonstrated high specificity, targeted delivery, and significant tumor reduction.

Another key factor of aptamers is that they are nontoxic. The advent of DNA origami and the creation of nanorobots has greatly transformed the field of targeted medicine delivery.^[^ ^238^, ^239^ ^]^ To treat tumor cells, Li et al. created nanorobots that were modified with AS_1411_ aptamer. The goal of this study was to enable technology to inject thrombin directly into tumor cells. This method led to a significant reduction in tumor size. Given the lack of toxicity, the translational potential, and the potential to design and construct increasingly advanced functionalities into nanorobots which might be programmed with multistep instructions, the delivery of CRISPR using DNA nanorobots and aptamers is an exciting approach for genome editing with a bright future.^[^ ^240^ ^]^

## 10 Enhanced CRISPR Delivery via Multicomponent NPs

While many synthesized NPs contain only one component, additional desirable characteristics can be obtained when the NPs comprise two or more different components, forming multicomponent NPs (MCNPs). MCNPs are nanoconstructs that combine the physical and biological properties of multiple materials into a single structure. Such combinations offer unparalleled opportunities for simultaneous diagnosis and treatment, known as theranostics, for a wide range of human diseases.^[^ ^241^ ^]^ By combining two or more components, the particles can acquire antimicrobial properties, chemical–mechanical polishing capabilities, imaging properties, and various other functions.^[^ ^242^ ^]^

The design of many components within a single platform, however, sometimes presents considerable difficulty and complexity in fabricating and characterizing these nanomaterials. It is essential to verify that NPs contain the right elements through nanocharacterization and that the inherent properties of the materials are preserved, or even improved, to provide the desired clinical benefits.^[^ ^243^, ^244^ ^]^ The characterization of NPs, including size, shape, surface charge, and porosity, is inextricably linked to their fabrication and ultimately, characterization ensures that the produced compounds have the appropriate characteristics and that batch properties are reproducible. Accurate and exact characterization is required to connect the physicochemical features of NPs with their performance in a particular function. The ability to evaluate characterization results and eventually manage the structure–function relationship of NPs can be greatly enhanced by understanding these connections.^[^ ^245^ ^]^

**Figure 4:**
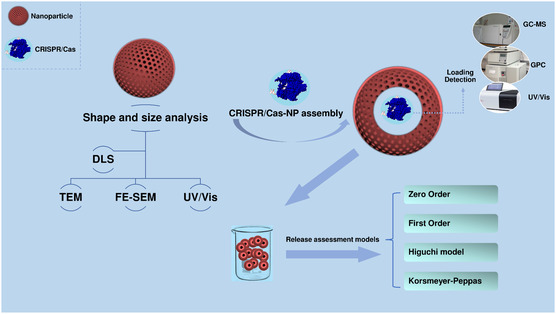
In the nanocharacterization workflow, ensuring the sufficient quality of NPs, size, and shape should be checked after their synthesis. Subsequently, the loading of CRISPR into the nanocarrier should be examined using the noted strategies. Finally, by employing the mentioned models, it is possible to assess the release of CRISPR from NPs.

Nanocharacterization can be performed in several ways (**Figure** **4**), and some examples of the more common methods are as follows. UV/vis spectroscopy can be applied to study, identify, and characterize various nanomaterials. The spectrum measured can be compared with predicted spectra using numerical models. The predetermined spectra (called standard curve) are determined for each material in pure form. Following the synthesis and combination of materials, and the production of NPs, measured spectra can be compared to the pure predicted standard curve.^[^ ^246^ ^]^ NPs exhibit optical properties depending on their shape, size, agglomeration state, concentration, and refractive index close to the NP surface.^[^ ^247^ ^]^ Another technique for NP characterization is transmission electron microscopy (TEM), an ultrahigh‐magnification measurement and visualization method that can image the NP size, grain size, size distribution, and morphology.^[^ ^248^, ^249^ ^]^ The samples are prepared for TEM imaging by drying them on grids, such as copper, that often have been coated with carbon. TEM can be utilized for metal NPs (e.g., gold, silver, aluminum, copper) and other NPs such as carbon nanotubes, magnetic, and polymeric NPs.^[^ ^250^ ^]^ In addition to TEM, scanning electron microscopy (SEM) and field emission SEM (FE‐SEM) are employed for related purposes.^[^ ^251^ ^]^

Dynamic light scattering (DLS) is another important and common technique for characterizing NPs and quantifying their properties. DLS measures the diameter of NPs based on the laser light scattered by NPs passing through a solution,^[^ ^252^ ^]^ and calculates the diameter using knowledge of the solution's properties and assumptions such as the spherical nature of NPs, to compute a Stokes radius. Hydrodynamic diameter is an important factor in other sizing measurement tools, such as TEM, because it gives information about the aggregation expression of NP solutions.^[^ ^253^ ^]^ Furthermore, Zeta potential, which determines the quantity of charge stability of NPs in colloids by measuring the effective electric charge on their surfaces, is another method for nanocharacterization.^[^ ^254^ ^]^

A critical step after identifying the shape, size, charge, and other characteristics is to assess the NP loading of the designed system (in our case, CRISPR). To accomplish this, several approaches have been developed. Gas chromatography–mass spectrometry (GC–MS) and gel permeation chromatography (GPC) are two well‐known technical methods for detecting loaded molecules. GC–MS analysis can detect even tiny amounts of a substance, while GPC can measure the molecular weight distribution of nanopolymers and particles loaded in polymer samples.^[^ ^255^ ^]^ The final step in nanocharacterization is release assay. The release of targeted components like CRISPR from NPs can be modeled and evaluated using various release models such as the Higuchi model, zero/first order, and Korsmeyer–Peppas model.^[^ ^256^ ^]^ During release assessment, the model with the greatest linearity will be used to determine the correct release rate.^[^ ^257^ ^]^ After verifying the quality of MCNPs, the transient delivery of CRISPR elements by MCNPs enables the transportation of various CRISPR formats, including a plasmid expressing the Cas9 endonuclease and gRNA, Cas9 mRNA plus gRNA, or tCas9/gRNA RNP complex.^[^ ^258^ ^]^

In **Table** **5**, we have listed some of the most common MCNPs and their applications, including liposome‐coated mesoporous silica NPs (lipoMSN), protamine and AuNPs, polyethylene glycol‐*b*‐poly lactide‐*co*‐glycolide‐based cationic lipid‐assisted NPs (PEG‐*b*‐PLGA‐based CLANs), lipid‐coated mesoporous silica NPs (LC‐MSN), lipid‐containing oligoaminoamides (lipo‐OAAs), and PEG‐poly(γ‐4‐((2‐(piperidin‐1‐1yl)ethyl)aminomethyle)benzyl‐l‐glutamate (PEG‐PPABLG). Using lipoMSN as a CRISPR delivery approach has been shown to be successful for multiplex gene editing in the mouse liver.^[^ ^258^ ^]^

**Table 5:** Multicomponent NPs designed for CRISPR delivery.

Type of MCNPa)–c)	Strategies for delivering CRISPR cargo	Target gene	Application	Disease	Properties	References
LC‐MSN	RNP complex and Cas9–gRNA plasmid	RFP	Edit genes in reporter cancer cell lines in vitro and in an Ai9‐tdTomato reporter mouse model in vivo	Cervical and lung cancer	Biocompatible and effective release inside cancer cells	[260]
LipoMSN	Cas9/gRNA RNP + Cas9 plasmid	pcsk9, apoc3, and angptl3	Synergistic effects on lipid metabolism, by combining targeted RNPs in NPs	Cardiovascular	An efficient delivery method for multiplex gene editing, providing a large surface area for the electrostatic loading of Cas9/gRNA RNP cargo with reduced charge density	[258]
CLAN	Plasmid encoding Cas9 mRNA and gRNA	CD40	Inhibition of T‐cell activation, which reduced graft damage and improved graft survival.	Reprogramming dendritic cells	Efficient delivery method into DCs	[164]
Protamine–AuNCs	Cas9–gRNA plasmid	EGFP and E7	Perform gene editing in cells and knock off the oncogenic gene for cancer treatment	Cervical cancer,	The cationic protamine encases the anionic DNA molecules in a compact manner, with nucleus‐targeting abilities and resistance to protease degradation	[112, 368]
Lipo‐OAAs	Encapsulating Cas9 protein and single gRNA	FolR1	Gene knockout, leading to an increase in nuclear association	Cervical and neuroblastoma cancers	High cellular uptake and high membrane lytic potential	[261]
LHNP	Cas9 protein and gRNA	PLK1	Inhibited tumor growth and improved the survival of tumor‐bearing mice	Brain tumors	A novel core–shell nanostructure, optimized for efficient codelivery of protein and nucleic acids, it penetrates the blood‐brain barrier	[175]
SMOF	RNP complex	BFP	Efficient genome editing within murine retinal pigment epithelial tissue is achieved	Variety of eye diseases	A pH‐responsive SMOF consisting of both silica and zeolitic imidazole framework, providing high loading content, excellent stability, and robust intracellular delivery of a variety of payloads, along with pH‐controlled release and endosomal escape capabilities	[265]
PEG‐PPABLG	Cas9–gRNA plasmid	PLK1	Conduct PLK1 gene knockout	liver cancer	Low cytotoxicity and high transfection efficiency	[261, 369]

a) FolR1, folate receptor 1 gene;

b) DNP, multistage delivery NP;

c) ZIF‐90, zeolitic imidazole framework‐90.

One successful MCNP used for CRISPR delivery is the liposome‐templated hydrogel NP (LHNP). An LHNP was developed to codeliver Cas9 protein and gRNA for inhibiting the PLK1 gene in tumors, including brain tumors. The LHNP core, which encapsulates the Cas9 protein, is a hydrogel formed by cyclodextrin‐PEI cross‐linking with adamantine‐PEI. The shell consisting of DOTAP is involved in the effective delivery of gRNA. The results demonstrated that delivery with LHNP effectively inhibited tumor growth (the average tumor volume in the LHNP treatment group was 23.5% of that in the control group) and improved the survival of tumor‐bearing mice (40 days for the LHNP treatment group compared to 29 days for the control group).^[^ ^175^ ^]^ Another report successfully delivered Cas9 protein and gRNA‐targeting PLK1 plasmid by a nanocarrier consisting of a metal core and an anionic lipid shell (1,2‐dioleoyl‐3‐trimethylammonium‐propane (DOTAP), 1,2‐dioleoyl‐sn‐glycero‐3‐phosphoethanolamine (DOPE), cholesterol, DSPE‐PEG). This NP reduced the expression of the PLK1 protein by more than 70% in the A375 cell line and inhibited melanoma progression by up to 75% in mice.^[^ ^113^ ^]^

Recently, Luo et al. synthesized cationic and amphiphilic lipid‐assisted PNPs. The primary aim of their study was to synthesize NPs for macrophage‐specific in vivo transfer of CRISPR for gene editing. This was achieved by constructing plasmids with a macrophage‐specific promoter that drives the expression of Cas9 (pM330 and pM458). This plasmid was further fused with gRNA that targets Netrin 1 (Ntn1) gene expression. In the next step, this construct was further fused with cationic lipid‐assisted PNPs. When these NPs containing the CRISPR plasmid were intravenously injected into mice, this construct successfully silenced the Ntn1 gene. This strategy effectively improved the symptoms of Type 2 diabetes mellitus, opening new possibilities for precise gene editing using the CRISPR system.^[^ ^259^ ^]^

The technique of PEG‐*b*‐PLGA‐based CLANs demonstrates how to reduce inflammation in the microenvironment by modulating immune cells directly using NPs carrying a payload of genome editing tools. LC‐MSN is a mesoporous silica NP delivery vehicle that is coated with lipid, which permits both loading (RNP or plasmid NPs) and effective release of CRISPR components into cancer cells.^[^ ^260^ ^]^ Lipo‐OAAs is another example of MCNPs that are very successful in delivering Cas9 and gRNA into cells and disrupting genes. Indeed, use of lipo‐OAA, including hydroxy‐stearic acid, is preferable to the use of analogs containing saturated or unsaturated fatty acids without hydroxylation because the approach creates smaller, more well‐defined NPs using Cas9/gRNA and improves cell absorption and endosomal release.^[^ ^261^ ^]^ The lipo‐OAAs assemble into NPs that are more defined and more miniature with Cas9/gRNA, increasing nuclear association and causing the maximum level of gene knockouts.

Yin et al. have successfully synthesized a cationic peptide, poly(γ‐4‐((2‐(piperidin‐1‐1yl)ethyl)aminomethyle)benzyl‐l‐glutamate) (PPABLG). PPABLG, being cationic in nature, possesses an alpha‐helical polymeric vector utilized for gene editing. To enhance its gene delivery efficiency, it was fused with PEG, resulting in the creation of PEG‐PPABLG. Consequently, NPs could traverse the plasma membrane and bypass endosomal degradation easily. When combined with PEG‐PPABLG, a plasmid expressing Cas9/gRNA achieved an expression level of up to 60 percent and efficiently knocked out the PLK1 gene.^[^ ^32^, ^262^ ^]^

MCNPs incorporating AuNPs also hold fascination. The properties of protamine and AuNPs are combined in protamine–AuNCs: the cationic protamine makes it easier for Cas9–gRNA plasmids to be released into the cell's nucleus. Protamines are highly cationic nuclear proteins that include a high percentage of arginine residues (up to 67%). It is widely known that cationic protamines tightly package anionic DNA molecules. It is widely known that cationic protamines tightly package anionic DNA molecules. Thus, protamines with cell‐penetrating and nucleus‐targeting capabilities have a high potential for efficient gene transport into cells. AuNCs can quickly assemble with Cas9–gRNA plasmids to enable efficient cellular delivery.^[^ ^112^ ^]^ Another intriguing MCNP was created by integrating Aptamer and AuNPs. In this regard, Tang et al. developed a nanoplatform consisting of gold nanorods modified with a TAT (GRKKRRQRRRPQ) and Linker‐Aptamer for coassembling the gRNA/Cas9 complex, successfully combining gene editing and photothermal therapy for tumors.^[^ ^263^ ^]^ Once the surface of AuNPs has been modified, CRISPR components can be loaded via electrostatic interactions. A study conducted by Wang et al.^[^ ^264^ ^]^ demonstrated that pCas9‐containing MCNPs (containing AuNPs) were able to suppressing tumor cell growth. Once taken up by cells, Cas9 immediately dissociates from the pCas9/AuNPs complex via a laser‐triggered photothermal effect. Furthermore, it was shown that a cell‐penetrating peptide such as cationic TAT peptide can facilitate the uptake of the pCas9/sgPLK1 complex into the nucleus, resulting in the silencing of the PLK1 gene and ultimately suppressing tumor development.

Silica–metal–organic particles have also been used to fabricate NPs for genetic drug delivery. For example, pH‐responsive SMOF NPs were fabricated to deliver various payloads containing hydrophobic small molecule drugs, nucleic acids, as well as genome‐editing machinery. This nanoplatform consists of silica and zeolitic imidazole framework, and its superiority in drug delivery/genome editing is attributable in part to the pH‐mediated release and endosomal escape on account of the proton sponge effect of imidazole moieties. The results demonstrated the induction of efficient genome editing using Cas9‐gRNA RNP‐loaded SMOF NPs in vivo within mouse retinal pigment epithelial tissue.^[^ ^265^ ^]^

## 11 CRISPR Delivery via Viral Vectors

CRISPR delivery systems include both viral and nonviral carriers. Choosing the appropriate delivery method for specific applications requires understanding the advantages and disadvantages of these different delivery strategies. An improved understanding of these specifications will moderate the challenges of delivering the CRISPR system for gene editing.^[^ ^266^ ^]^ Due to the fact that genetic diseases only affect a subset of tissues and organs, the gene or cell delivery must specifically target that region without degradation, and off‐target effects can result in toxic outcomes as well as death. Moreover, the large size of CRISPR‐related systems may limit their use for some NP systems due to an inability to properly load the cargo, without impacting the NPs’ key physicochemical characteristics. Thus, targeting gene transfer vectors in a spatially and temporally controlled manner to tissues and organs can be challenging. In the case of CRISPR, the host's immune response is another important issue.^[^ ^267^ ^]^ In vivo CRISPR therapies showed human immune response against Cas9 proteins may produce undesirable outcomes and side effects. So, understanding Cas9's immunogenicity is crucial to developing future therapeutics.^[^ ^268^ ^]^

Since the 1980s, scientists have been interested in viruses’ natural ability to transduce cells and tissues containing foreign nucleic acids as a means of gene transfer.^[^ ^269^ ^]^ Schmidt and Grimm, who investigated viral vectors to transmit CRISPR extensively in 2015, suggested that there would not be one viral vector system to address all applications. Several factors must be considered, such as 1) integration capabilities: the capacity to integrate into the host genome, 2) packaging capacity: the maximum size of genes within a package, 3) carrier specificity: related to the viruses’ ability to recognize and bind specific molecules on the surface of cells which causes cell selectivity (vector tropism), 4) safety: refers to the particular environments under which they may be produced and handled, as well as their effects on treated cells, and 5) immunity response: host immune response to the viral vector.^[^ ^270^, ^271^, ^272^ ^]^ So far, many viral vector classes have been developed and tested as Cas9 and/or gRNA delivery vehicles, including retroviruses, lentiviruses, adenoviruses, and AAVs.^[^ ^270^, ^273^ ^]^ We discuss the characteristics of these viral vectors and compare the key features related to CRISPR applications.

### 11.1 Retroviral Vectors

The following features of retroviral vectors contribute to their use as a transfer of genetic information in gene therapy: 1) incorporation of membrane‐coated virus particles into target cells via receptors; 2) an infection caused by reverse transcription of a plus‐stranded RNA genome to a double‐stranded DNA that is integrated into a cell's chromosome; and 3) assembly of particles incorporating full‐length retroviral mRNA as the type of genetic information mobile within the cytoplasm.^[^ ^274^, ^275^ ^]^ In addition, the envelope protein of retroviruses can be replaced with the glycoprotein of other viruses to achieve pseudotype, enabling the pseudovirus to infect different target cells.^[^ ^276^ ^]^ Gamma‐retroviruses (γ‐RVs), such as the Moloney murine leukemia virus (M‐MLV), and lentiviruses, including the human immunodeficiency virus type 1 (HIV‐1), are among the most common retroviruses considered in gene therapy because they can be engineered to carry transgenic alterations. As noted above, they can be stably integrated into host chromosomes and thus lend themselves to long‐term expression in gene therapy.^[^ ^277^ ^]^ It is a technological breakthrough to use retroviral gene therapy for the delivery of genome editing tools that combine efficient gene transfer and site‐specific genetic modification potential. By harnessing the power of these molecular tools in a safe manner, CRISPR is likely to further improve many therapies and possibly eliminate a number of diseases.^[^ ^278^ ^]^

### 11.2 Lentiviral Vectors

Lentiviruses, belonging to the retrovirus family, contain single‐stranded RNA genomes (ssRNA) ≈10.7 KB long and enclosed in a fat‐enriched spherical capsid. The lentiviral genome contains three essential genes: 1) gag: which encodes structural proteins for virion assembly and infection, 2) pol: which encodes enzymatic proteins for reverse transcription and integration into the genome, and 3) env: which encodes the viral envelope glycoprotein for binding to cellular receptors.^[^ ^53^ ^]^ Lentiviruses were originally derived from HIV‐1; however, their host tropism has been altered by incorporating numerous heterologous envelope glycoproteins during viral assembly. Lentiviruses take advantage of the host‐protein machinery to efficiently cross the nuclear membrane. As a result, these viruses have been engineered and become useful, effective, and capable vectors of transduction into nondividing or dividing cells.^[^ ^279^, ^280^ ^]^

In addition, lentiviral vectors have become valuable tools for genome editing and delivering CRISPR components because they can carry large and complex transgenes; they can also maintain robust, long‐term expression across a wide range of cell types, depending on whether they are dividing or nondividing.^[^ ^53^, ^281^ ^]^ Lentiviral classification is based on integrating or nonintegrating lentiviral vectors (NILVs). Stable integration into the genome remains a potential concern with the use of integrating lentiviral vectors. Therefore, NILVs are preferred.^[^ ^282^ ^]^ Based on CRISPR system variants, lentiviral CRISPR vectors can also disrupt noncoding RNA expression, such as miRNA genes.^[^ ^283^ ^]^

Despite their usefulness as CRISPR delivery systems for preclinical studies, achieving stable expression of CRISPR constructs and conducting powerful CRISPR‐based screens, LVs, like all integrating viruses, pose a risk of insertional mutagenesis, in addition to the risk of sustained expression of CRISPR/Cas which may result in off‐target mutations and genomic instability.^[^ ^284^ ^]^ To address these limitations nonintegrating or integrase‐deficient lentivirus vectors have been recently engineered with therapeutic purposes in mind and, they share the following characteristics: 1) transmittance to a diverse range of tissues and cells; 2) higher packing capacity than other vectors; 3) transient expression and very poor integration ability; and 4) low immunogenicity.^[^ ^54^, ^282^, ^285^, ^286^ ^]^

### 11.3 Adenoviral Vectors

The adenovirus is an uncoated double‐stranded DNA virus that has a 36 KB genome, and does not integrate into the host genome, eliminating the risk of carcinogenicity or gene toxicity associated with other integrating vectors. Hexon, panton, and fiber are the three main proteins in the capsid surrounding the genome, and all three are known to resist genetic modification. By using the adenovirus packaging system, high‐quality recombinant viruses and high‐target gene expression can be produced. Moreover, these vectors are compatible with the new genome‐editing system CRISPR. Due to these features, adenoviruses are an attractive candidate for genome editing using CRISPR because they have been proven safe in clinical trials.^[^ ^55^, ^287^ ^]^

Aside from the above, this category of viruses has some general advantages, including genetic stability, well‐defined biology, placing large and high titers with little or no insertion mutagenesis; and disadvantages, such as ubiquitous tropism and significant immunogenicity and more difficult production.^[^ ^288^ ^]^ There are three different generations of adenoviruses with different characteristics, among which helper‐dependent adenovirus vectors, belonging to the third generation, has been the type considered for use with CRISPR systems have been considered for genome editing. The advantages of high‐capacity AdVs (HCAds) include a large packaging capacity allowing for all CRISPR components to be incorporated into a single vector, and lack of all viral genes, so no viral gene expression can occur.^[^ ^289^ ^]^ Less immunogenicity and the adaptability of the CRISPR system are additional benefits of HCAds vectors. Airway delivery of CRISPR/Cas9 with this platform shows great potential for lung gene therapy.^[^ ^290^, ^291^ ^]^

### 11.4 AAV‐Based Delivery Platforms

AAVs is one of the most commonly used viruses in clinical‐stage delivering CRISPR.^[^ ^292^ ^]^ AAVs are nonenveloped viruses with highly infectious, display mild immunogenicity, and have high in vivo tolerability, do not normally integrate into human DNA, and only 4.7–5 KB of packing capacity is available, so they have attracted a lot of attention, especially in clinical‐stage treatments.^[^ ^293^, ^294^ ^]^ The AAV genome is made up of a single‐stranded DNA.^[^ ^295^, ^296^ ^]^ As AAV/CRISPR vectors have low immunogenicity, their handling does not require special precautions. The vector can also be used for cells that are resistant to DNA transfection, thereby extending the areas to which CRISPR can be used.^[^ ^297^ ^]^ Recently, recombinant adeno‐associated virus (rAAV) has been considered for gene transfer, so many groups are focusing their efforts on using this tool to deliver compact Cas9 orthologs in vivo. Most efforts to edit the Cas9 genome have focused on the widely used ortholog type II‐A of *S. pyogenes*, SpCas9.^[^ ^51^, ^298^ ^]^ SpCas9 is a CRISPR self‐limiting system that shortens the Cas9 expression time by having recognition sites on its expression cassette.^[^ ^299^ ^]^ With a smaller orthologue, *Staphylococcus aureus* (SaCas9, a single AAV vector), incorporates both SaCas9‐ and gRNA‐expression cassettes as a single vector with high titer expression.^[^ ^300^ ^]^ There are many examples of AAV used with CRISPR for gene therapy listing some here can be helpful. AAV is also used to deliver HDR templates for precise CRISPR editing, and is a very useful way to bypass the need for delivery of DNA donor templates in addition to the CRISPR components. Like adenoviruses, AAV has the potential specific use for pulmonary gene therapy.^[^ ^301^ ^]^ **Table** **6** summarizes different types of viral vectors developed for CRISPR delivery along with many of their key characteristics.

**Table 6:** CRISPR viral vectors and their key characteristics.

	Retroviruses		Adenoviruses	AAVs
	Nonlentiviruses	Lentiviruses		
Genome integrating	✓	✓	×	×
	Stabilize their genetic information on host chromosomes	Load large amounts of DNA	Facilitate gene transfer in the absence of genome integration	Efficient long‐term gene transfer without integration
	Fusion of heterologous envelope proteins to modify target cell specificity	Broad expression in dividing and nondividing cells	Large cargo size	Wide host‐cell range
Packaging capacities [KB]	7–8	8–10	6	5
Cytotoxicity	Low	Low	Low	Low
Immunogenicity	Low	Low	strong	Weak
Genome structure	ssRNA	ssRNA	dsDNA	ssDNA
Reference	[370, 371]	[372, 373]	[374, 375]	[376, 377]

Overall, it is important to note that viral vectors have limited applications due to their restricted package size, which confines their use to smaller variants of CRISPR/Cas9 constructs.^[^ ^33^ ^]^ Furthermore, concerns related to immunogenicity prevent their repeated use in the same host. In addition to their limited capacity and high risk of adverse events, viral vectors suffer from sustained Cas9 expression, anti‐Cas9 immune responses, and off‐target editing.^[^ ^302^, ^303^ ^]^ For these reasons, researchers are exploring safer and less immunogenic alternatives for CRISPR/Cas9 delivery.^[^ ^37^ ^]^ Nonviral delivery systems offer reliable stability and delivery efficiency in vitro, ex vivo, and in vivo applications. They are less toxic and provide sustainable gene expression without causing unwanted inflammatory or immune reactions, making these systems a potent alternative to viral vectors.^[^ ^303^ ^]^

## 12 Enhancing CRISPR Functionality with VLPs

As mentioned above viral vectors have many limitations. Therefore, the creation of substitute vehicles is crucial due to the major drawbacks of viral vectors. VLPs were developed as a new delivery vehicle to overcome the challenges associated with the viral vectors and are used to improve CRISPR functions. VLPs embody viral proteins capable of infecting cells without carrying virus genetic material. They mimic the viral ability to enclose and protect genetic material from damage caused by nucleases.^[^ ^304^, ^305^, ^306^, ^307^ ^]^ Besides their low production cost and ease of handling, VLPs are also stable during maintenance and high in safety.^[^ ^308^ ^]^ VLPs combine the high transduction efficiency of viral‐derived vectors with the expected safety benefits of ultrashort expression of CRISPR/Cas components.^[^ ^309^ ^]^ Traditional viral vectors, such as lentiviruses and adenoviruses, contain viral genetic material and can provoke strong immune responses, leading to the production of neutralizing antibodies and T‐cell activation.^[^ ^310^, ^311^ ^]^ In contrast, VLPs self‐assemble into particles without containing viral genetic material, and their rapid clearance significantly reduces the risk of sustained immune activation and insertional mutagenesis. Furthermore, modifying the structure of VLPs through chemical engineering and mutagenesis can further reduce their immunogenicity.^[^ ^35^, ^305^, ^312^, ^313^ ^]^

VLPs can moreover be used to package SpCas9 RNPs.^[^ ^57^ ^]^ In addition, there are engineered VLPs that commercially serve as a standard or control in nucleic acid‐based diagnostic tests, as well as antigen epitopes in serological tests where patient antibodies are used for diagnosis.^[^ ^314^ ^]^ So far, several types of VLP designed to increase CRISPR system efficiencies.^[^ ^315^, ^316^, ^317^ ^]^ Qazi et al. showed that VLPs obtained from bacteriophage P22 could be generated in *E. coli* through the coexpression of coating protein and scaffold protein (SP). They developed a programmable delivery system by fusing Cas9 with P22, a SP that forms a protein capping to enclose Cas9. The objective of the experiment was to demonstrate the genetic flexibility of SP‐directed encapsulation, which presents new possibilities for fusing cargoes to either one of the SP terminal ends.^[^ ^318^ ^]^

The peptides used to make pVLPs are generally nonviral synthetic peptides and do not fully mimic virus structural function. Due to their cellular/nuclear‐penetration ability, biocompatibility, and capability to protect gene cargo from degrading, pVLPs that are coassembled from dual‐origin viral peptides and DNA tend to be more advantageous than other VLPs. CRISPR payloads can be delivered to the cell efficiently and with better fidelity through this method, possibly due to its intrinsic cell‐penetrating function.^[^ ^319^, ^320^ ^]^

Gene editing using CRISPR is generally concerned with off‐target effects that may occur at sites with sequencing that are highly similar to the target protospacer sequence. One approach to reducing off‐targeting effects is by using nanoblades, which deliver the Cas9‐gRNA complex transiently and dose‐dependently. Nanoblades (refers to CRISPR genome cutting ability) are murine leukemia VLPs that transport Cas9 RNPs. The nanoblades are tiny DNA‐cutting tools with very large surface areas that can be used to deliver CRISPR to multiple targets, including primary cells, embryos, and animals. By programming nanoblades with modified Cas9 protein, targeted genes can be transiently activated.^[^ ^317^, ^321^ ^]^

CRISPR VLN, another type of VLP, is being examined as a versatile platform for simultaneously delivering small molecular drugs and the CRISPR system for potentially treating malignant cancer. They have a core‐shell composition that is primarily enclosed in lipids. Moreover, a core comprises mesoporous silica NPs (MSN), which are used to load small molecule drugs and the CRISPR system. As a result of this structure, VLN remains stable while circulating in the blood. Thus, VLNs comprise an effective platform for the development of advanced combination therapies against malignancies because they are sufficiently versatile to enable delivery of nearly any combination of gRNAs and small molecule drugs to cancer.^[^ ^322^ ^]^

## 13 Exosomes for Delivering CRISPR

**Figure 5:**
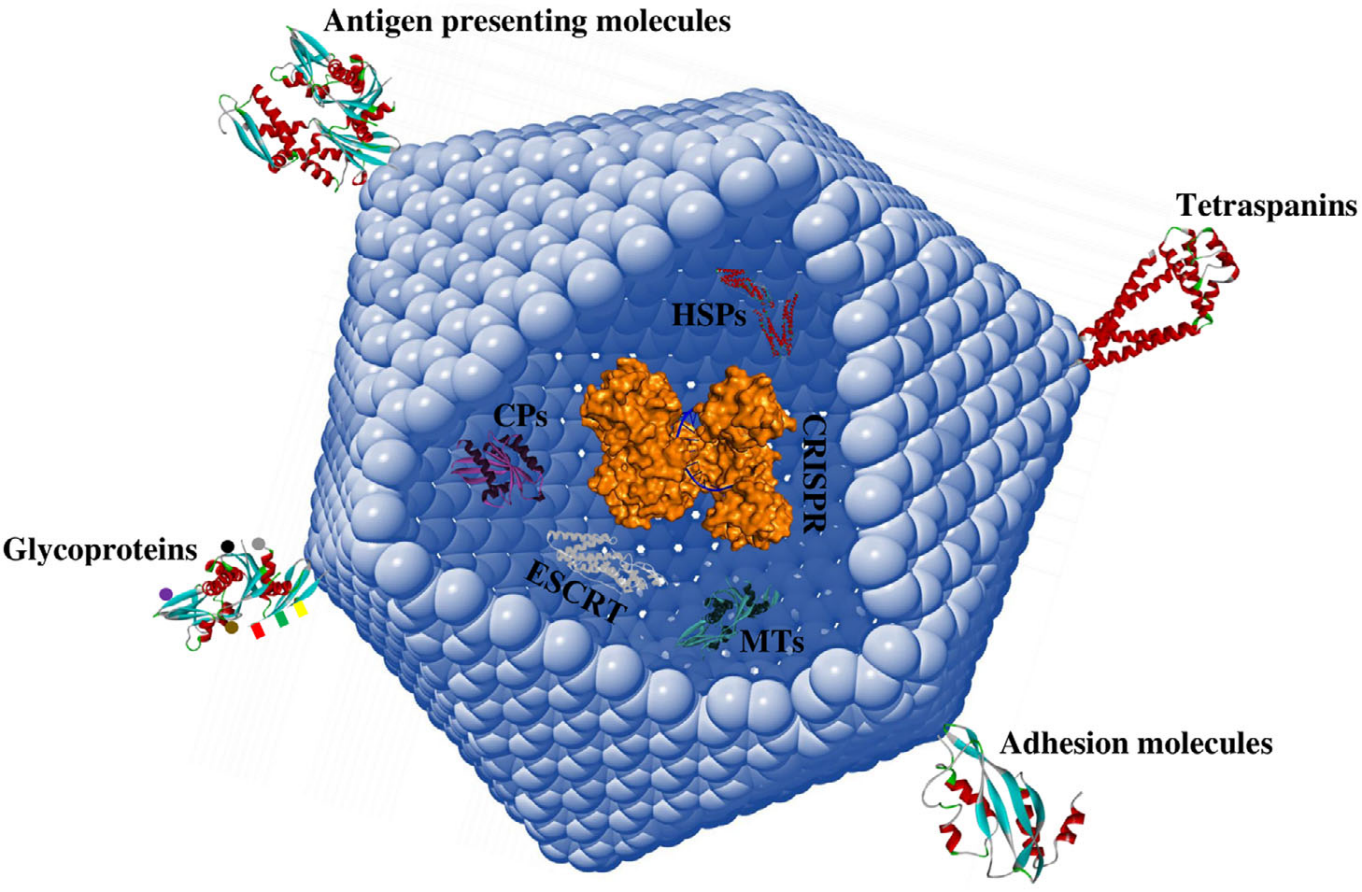
Exosome loaded with CRISPR platform for enhanced genome editing capabilities. Because of their bioavailability and biocompatibility, exosomes can be considered good carriers of CRISPR. Membrane transporters (MTs), heat shock proteins (HSPs), cytoskeletal proteins (CPs), and the endosomal sorting complex required for transport (ESCRT).

Recently, a new class of delivery system has emerged. Exosomes are membrane‐bound vesicles secreted by mostly all cell types, with sizes ≈30–150 nm.^[^ ^323^ ^]^ Owing to their intrinsic properties in cargo delivery including, prolonged circulating half‐time, minimal immunogenicity, easy handling, high payload capacity, blood–brain barriers passing potential, and foremost biocompatibility, they are privileged against other available delivery platforms. In this way, exosomes could be considered a potential vehicle for CRISPR/Cas (**Figure** **5**).^[^ ^324^, ^325^ ^]^

Researchers have been attempting to deliver CRISPR/Cas for genome editing in different diseases. In a study, He et al. engineered HEK293 cells to express Cas9 protein inside exosomes; after it, they electroporated the gRNA plasmid into the purified exosomes. The gRNA was designed to direct Cas9 protein to a specific genomic locus, the outcome was brilliant; this system could induce significant gene editing in HuH‐7 cells and diminish cancerous cell viability. Therefore, this experiment proposed a safe and efficient delivery approach for CRISPR/Cas9‐based gene therapy in liver cancer.^[^ ^325^, ^326^ ^]^ Since, due to the limitation of electroporation for large constructs including plasmids, novel approaches are developed.^[^ ^327^ ^]^

Exosome delivery of microRNAs and mRNAs was achieved by loading mRNA of Cas9 protein and the target gRNA sequence by electroporation into RBC‐derived vesicles. The target was the miR‐125 family locus with a potential mutation site. MOLM‐13 cells were treated with RBC‐derived exosomes which contain Cas9 mRNA and 125b‐gRNA, and gene editing was nearly perfect, resulting in 98% and 90% reduction in expression of miR‐125b and miR‐125a, respectively, after 48 h. The test was also conducted by Cas9 and GFP gRNA plasmids, but the efficiency proved worse than the mRNA method due to the larger size of the plasmid in comparison with mRNA. Thus, owing to the advantages of RBC exosomes, including their availability in blood banks (providing large‐scale sources of exosomes), higher payload capacity, and their safety due to DNA depletion, they are becoming ideal vehicles for CRISPR/Cas system delivery for cancer treatment.^[^ ^328^ ^]^ Delivering Cas9 and gRNA mRNA results in a more rapid response to CRISPR/Cas9 because of translation in the cytosol without needing to enter into the nucleus; moreover, the mRNA does not have genome insertion risk. Concerning the abovementioned advantages, degradability, and shorter half‐time still are issues that could be solved to some extent by exosome delivery.

The CRISPR/Cas system also could be delivered as an RNP by exosomes. Zhuang et al. designed an experiment to evaluate delivering capacity of CRISPR/Cas RNP for gene editing in vitro and in vivo models by exosomes. They treated HepG2/GFP cell line with Exosomes containing Cas9 and GFP‐gRNA complex, the GFP knockdown was significant. The exosome formulation showed a better GFP knockdown capacity in comparison with the liposomal formulation, the other delivery system. The same data were achieved in the HepG2 xenograft mouse model; several days after the establishment of HepG2 tumor‐bearing female BALB/c nude mice, the mice intravenously were injected with different formulations. Exosomal formulation demonstrated a higher potential in reducing WNT10B gene expression and tumor size in comparison with liposomal formulation.^[^ ^329^ ^]^ Using exosomes for delivering CRISPR/Cas system is not only limited to cancers. Exosomes are used to deliver CRISPR/Cas compartments in diverse diseases including Muscle atrophy,^[^ ^330^ ^]^ hepatitis B,^[^ ^331^ ^]^ DMD,^[^ ^332^ ^]^ and liver disease.^[^ ^333^ ^]^

While natural exosomes display inherent targeting at the cellular and tissue levels, this is often insufficient for targeted delivery.^[^ ^334^ ^]^ Engineered exosomes, developed through producer cell engineering, direct vesicle modification, and VLP engineering, enhance targeted delivery, reduce CRISPR off‐target effects, extend circulation time, improve cellular uptake, and promote lysosomal escape.^[^ ^35^ ^]^ Consequently, engineered exosomes have the potential to surpass the capabilities of natural exosomes in efficiently delivering CRISPR systems, thereby advancing their application in clinical settings.

Despite the various advantages of exosomes for delivering CRISPR/Cas systems, they are limited by their heterogeneity, low production yield, difficulties in isolation and purification, and inadequate targeted delivery. However, several strategies have been developed to address these limitations.^[^ ^335^ ^]^ Scalable production of exosomes could be achieved using bioreactors,^[^ ^336^ ^]^ and synthetic exosome generation could mitigate yield issues.^[^ ^337^ ^]^ Furthermore, various isolation and purification techniques, such as density gradient ultracentrifugation, ultrafiltration, size exclusion chromatography, and immunoisolation, can alleviate challenges associated with exosome isolation and purification.^[^ ^338^, ^339^ ^]^

## 14 Localized CRISPR/Cas Delivery

**Figure 6:**
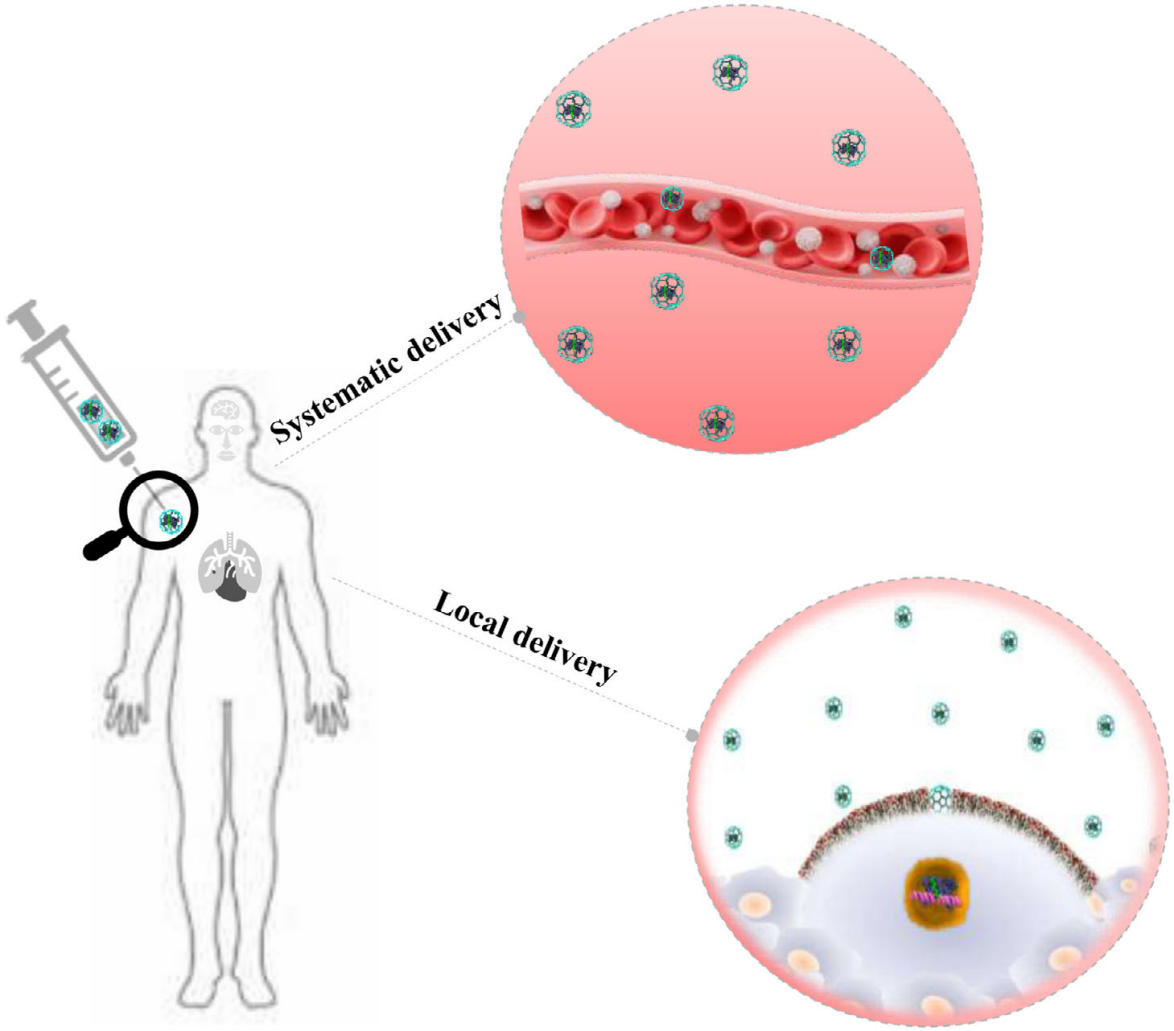
Systemic and local delivery of CRISPR involves different approaches. Local administration entails injecting directly into target tissues, whereas systemic administration involves injecting directly into blood vessels, allowing for a more homogeneous distribution throughout the entire body.

We have focused our discussion thus far mainly on the systemic delivery of CRISPR/Cas, given that most applications require such administration; however, under certain conditions, one can increase the efficiency and reduce limitations of the systemic approach using local delivery. In CRISPR/Cas local administration, the CRISPR/Cas system reaches an effective concentration with minimal injection; in addition, the system affects only specific organ or target site which reduce the off‐target effects (**Figure** **6**). Therefore, owing to the advantages of this approach, strategies for using local CRISPR/Cas delivery have increased.^[^ ^340^, ^341^ ^]^

In a study, Lee et al. designed and developed a gold NP‐based delivery system for the delivery of Cas9 RNP and donor DNA to treat the DMD mice model. Interestingly, after one dose of intracranial injection of CRISPR‐Gold, 5.4% of the mutated dystrophin gene was corrected and returned to the wild type by homology‐directed DNA repair. Surprisingly, when the Cas9 RNP and donor DNA were injected without nanoformulation, the correction rate of the gene was only 0.3%. Furthermore, the CRISPR‐Gold formulation did not result in acute up‐regulation of inflammatory cytokines in plasma and is considered a safe formulation.^[^ ^342^ ^]^ Therefore, gold NPs are an ideal delivery carrier for the local delivery of CRISPR/Cas elements.

A lipid NP entitled 8‐O14B was developed for delivery of Cas9/gRNA complex to the brain of the Rosa26^tdTomato^ mouse model. This mouse model disabled of transcription of red fluorescent protein (tdTomato), so successfully delivering Cas9/gRNA could result in tdTomato expression. Therefore, the nanoformulation was injected into different parts of the mouse brain and after several days the results were revealed. The outcome was compelling, the number of positive tdTomato cells was about 350 cells in a 0.5 mm^2^ area at the injection site. On the other hand, free injection of Cas9/gRNA could not reach the target cells and the tdTomato gene remained inactive. Consequently, the data confirmed that the dispersion of payload in the injected site was minimal, in this way, this approach provided a minimal off‐target and could be used as a suitable candidate for delivering CRISPR/Cas to the brain.^[^ ^204^ ^]^

Chen et al. developed another type of nanocarrier for the local delivery of CRISPR/Cas RNP. They synthesized a thin glutathione (GSH)‐cleavable covalently cross‐linked polymer coating, called a nanocapsule (NC). They tested the NC editing capacity in vivo in the eyes and muscles of transgenic Ai14 mice. The targeting gene was tdTomato fluorescent reporter like the aforementioned study. The mice subretinally were injected, and the result was impressive; a decorated formulation of NC, NC‐ATRA (all‐trans retinoic acid) showed a robust editing potency in comparison with NC and free RNP. In addition, muscle injection with NC also revealed better editing ability compared to free RNP.^[^ ^343^ ^]^ The data suggest that using nanocarriers is a promising approach for efficient local delivery of CRISPR/Cas into the target sites, with minimal cytotoxicity.

Recently, a novel nanovector was developed by Choe et al. for the local delivery of the CRISPR/Cas system for the treatment of X‐linked juvenile retinoschisis (XLRS). This nanovector entitled supramolecular NP (SNMP), which was synthesized by mixing three molecular building blocks, β‐cyclodextrin (CD)‐grafted branched polyethyleneimine (CD‐PEI), adamantane (Ad)‐grafted polyamidoamine dendrimer (Ad‐PAMAM), and Ad‐grafted poly (ethylene glycol) (Ad‐PEG).^[^ ^344^, ^345^ ^]^ In this experiment, two SNMP were synthesized for codelivery of Cas9/gRNA‐plasmid and Donor‐RS1/GFP‐plasmid for CRISPR/Cas9‐mediated knocking of RS1 gene at the Rosa26 site in the mouse retinas. In this way, two SNMP vectors that carry Cas9/gRNA‐plasmid and Donor‐RS1/GFP‐plasmid were injected intravitreally into the Balb/c mouse eyes. After several days the injected site was analyzed by optical, pathological, and molecular tests. The outcome was impressive, CRISPR/Cas9‐mediated knocking of 3.0‐kb RS1/GFP gene into the Rosa26 site in mice retinas was confirmed, and delivery of CRISPR/Cas by SNMP vectors was successful. Furthermore, several predicted sites for off‐target integration were assessed; interestingly, no integration was found in predicted sites with molecular and optical tests. Consequently, this efficient delivery system with minimal off‐target was proposed for local delivery of CRISPR/Cas to treat XLRS.^[^ ^346^ ^]^

Local administration of the CRISPR/Cas system is a desired approach. This system could bring sufficient clinical benefits to patients by repairing the particular portion of cells in target tissues, with minimal safety risk. Several in vivo studies confirmed that local delivery of CRISPR/Cas with nanocarriers could result in promising outcomes, including in the brain,^[^ ^204^ ^]^ muscle,^[^ ^347^ ^]^ and eye.^[^ ^343^, ^346^ ^]^ Even though local delivery has advantages but still other items including the disease state, severity, properties of the protein encoded by the target gene, and the desired tissue are elements that should be considered for the successful delivery of the CRISPR/Cas system.^[^ ^348^ ^]^ Hopefully, recent studies paved the way for developing novel and efficient carriers for delivering CRISPR/Cas system. We should know that owing to the advantage of local delivery, this option should be mutually considered as a way for diseases which routinely treated by a systematic approach.

## 15 Engineering Carriers for Immune Cell Manipulation Using CRISPR Technology

Immunotherapy is a recent therapeutic pillar within the last 1–2 decades that imposes its effects by modulating the immune system, generally either by activating or inhibiting the immune response. This treatment strategy, which includes checkpoint inhibitors, vaccines, and cell‐based therapies, has been established to increase treatment efficiency, especially in cancers. Increased understanding of the role of the immune system in disease pathogenesis is resulting in the testing of novel immunotherapies for a variety of other diseases, such as atherosclerosis.^[^ ^349^ ^]^ T‐cells and natural killer (NK) cells are often considered the most critical effector cells in the immune response; therefore, modulating (or “engineering”) these cells could provide significant advantages in recognizing tumor cell antigens and treatment of a broader range of cancerous cells.^[^ ^350^, ^351^ ^]^

Recent developments in genome editing tools have introduced a novel gate for immunotherapy. Nowadays, CRISPR/Cas is the prevalent editing system and provides many advantages for immunotherapy. CARs (chimeric antigen receptors), including CAR‐based NK and T‐cell therapy, have shown significant clinical outcomes in cancer patients.^[^ ^352^ ^]^ However, safe, effective delivery of CRISPR/Cas system in vitro, ex vivo, and in vivo remains challenging.^[^ ^353^, ^354^ ^]^ Nano‐based delivery systems could be suitable for handling these challenges. Nanocarriers have been well developed to deliver their payload to specific immune cells by decorating their surface with targeting agents, which significantly could minimize the off‐targeting of the CRISPR/Cas system. In addition, electroporation is a well‐established method for delivering this system to the cells, which could be manipulated outside the body. Still, it does not apply to inside‐body delivery. Therefore, different nanocarriers are present, which could engulf diverse payloads like CRISPR/Cas system and preserve their cargo from degradation until reaching the target site in in vivo studies.^[^ ^353^ ^]^ The use of nanocarriers to efficiently deliver CRISPR/Cas systems to immune cells for immunoengineering is a natural extension at the intersection of immunology, nanotechnology, and gene editing and given nanotechnology's advantages.

Recently, a Cas9 RNP and CAR transgene were successfully delivered to edit CD4^+^ cells in mixed T‐cells using engineered VLPs. This system demonstrated a novel platform for simultaneous, cell type‐specific genome editing and transgene introduction. Briefly, Cas9 protein was fused to the lentiviral Gag structural protein for successful packaging in VLP, producing a carrier containing lentiviral‐encoded CAR and Cas9 RNP complex. This codelivery system was highly efficient in lentiviral genome integration and Cas9‐mediated knockout. Finally, specific delivery of this system to CD4^+^ T‐cells in a cell mixture was achieved using a viral pseudotyping with a viral glycoprotein, the HIV‐1 viral glycoprotein Env.^[^ ^353^ ^]^ This approach, including borrowing from biology to power efficient, specific cell delivery, could pave the way toward engineering specific immune cells in vivo, including as a promising strategy for cancer nanoimmunotherapy.

A novel, highly efficient, and safe nonviral protocol for CRISPR/Cas9‐mediated gene editing of human T‐cells by electroporating a plasmid donor DNA template and Cas9‐RNP. The donor template inserted a CAR construct into the T‐cell receptor α constant (TRAC) locus within the pUC57 plasmid (nanoplasmid). This method significantly increased the genomic integration of T‐cells compared to the free dsDNA template at similar dosages because linear dsDNA is toxic to T‐cells in higher concentrations, thereby increasing the engineering efficiency of such cells.^[^ ^355^ ^]^ These increases in safety suggest the approach should be further explored for translational clinical studies.

Despite the temptation to focus on them, engineering T‐cells and NK cells are not the only options for cancer immunotherapy. Indeed, it is becoming increasingly clear that given the complex cellular milieu involved in the pathogenesis of disease, multiple cell types may need to be therapeutically engineered to produce significant clinical responses in a large fraction of the population. For example, while activating one cell type, such as T‐cells, it might be concurrently necessary to repress immunosuppressive cells, such as T‐regulatory cells, in order to yield significant effects. One method to reverse immunosuppression involves metabolic engineering of the tumor microenvironment as a promising strategy for cancer immunotherapy.^[^ ^356^ ^]^ Tumor cells display abnormal metabolic activity, including high glucose uptake and excess lactate production, which may result in immunosuppression.^[^ ^357^, ^358^ ^]^

A lipid NP‐based system for CRISPR/Cas9‐mediated metabolic engineering was developed to target the lactate dehydrogenase A (LDHA) gene to decrease tumor cell lactate production. Lipid NP‐loaded plasmid DNA coencoding CRISPR/Cas9 and sgLDHA was tested in vitro, showing editing of the B16F10 cell metabolic pathway by diminishing lactate production with no significant off‐target impact compared to the untreated group. To connect modulation of lactate concentrations with immunotherapy, the effect of lactate on T‐cell activity was tested in vitro. CD3^+^/IFN‐γ^+^ and CD3^+^/granzyme B^+^ and T‐cell populations increased in the nano‐CRISPR/Cas9‐sgLDHA‐treated group, suggesting the association with the antitumor activity of T‐cells. Moreover, an in vivo study with the B16F10 tumor model also proved this system's efficiency by decreasing tumor size in a mouse model due to increased T‐cell activity.^[^ ^189,189^ ^]^

When body organs reach failure, sometimes the only option is organ transplantation. The immune system is critical in the rejection of the newly transplanted organ, and T‐cells are key players in this reaction. But direct T‐cell targeting may not be the most efficient method to control rejection; instead, modulating immune cells that control effector cells provides a greater degree of control. For instance, T‐cell activation could be reduced by modulating dendritic cells (DCs), e.g., by inhibiting the costimulatory signaling molecule CD40 of DCs.^[^ ^359^ ^]^ Accordingly, a CRISPR/Cas9 gene‐editing system was targeted to the costimulatory molecule CD40 in DCs by loading Cas9 mRNA and gCD40 in poly(ethylene glycol)‐*block*‐poly(lactide‐*co*‐glycolide) (PEG‐*b*‐PLGA)‐based cationic lipid‐assisted NPs termed CLANmCas9/gCD40. This system was evaluated in in vitro and in vivo studies and successfully inhibited the costimulatory molecule CD40 in DCs. In the mouse model, IV injection of CLANmCas9/gCD40 resulted in the inhibition of T‐cell activation by reducing the expression of CD40 and, consequently, diminished graft damage and extended graft survival.^[^ ^164^ ^]^ Therefore, by controlling the response of specific immune populations under certain conditions such as organ transplant, it is clear that nanomedicine has opened a new window for the efficient delivery of CRISPR/Cas to better manage disease and other inflammatory conditions. Indeed, needed improvements in the specificity of nanomaterials for certain immune cell subsets, improving delivery efficiency,^[^ ^360^, ^361^, ^362^ ^]^ is perhaps the critical obstacle in the field of nanoimmunoengineering using CRISPR. By increasing the selectivity, not only will therapeutic efficacy improve, but critically the risk of adverse effects will correspondingly decrease, diminishing potential toxicities of the nanocarrier strategy and resulting in a more straightforward clinical path.

## 16 Conclusion and Outlook

The CRISPR technology offers a multitude of applications, including the generation of cellular and animal models with desired features (such as models that are more resistant to disease or have a specific gene expression pattern), improving fuel and food production, facilitating pathogen detection, accelerating drug development (e.g., high‐throughput drug screening), and advancing gene therapy. The expansion of CRISPR delivery platforms signifies a monumental leap forward in genome editing, providing researchers with powerful tools to combat diseases through precise editing of cellular genetic material. This symbiotic relationship between delivery platforms and CRISPR‐based genome editing not only enhances efficiency but also precision, propelling the field into uncharted realms of therapeutic potential.

Over time, an array of carrier classes, both bio and synthetic, has been developed to facilitate in vivo delivery of CRISPR components. Among these carriers, NPs, viral particles, VLPs, and exosomes have emerged as frontrunners due to their innate properties conducive to efficient cargo transportation. While these carriers have provided increased safety, lower cost, and higher efficiency for RNP administration, ongoing efforts aim to refine their performance for in vivo applications. These efforts focus on critical parameters such as biocompatibility, biodegradability, induction of immunogenic and inflammatory responses, off‐target effects, low stability, and disruption of engineered sequences due to unwanted genetic mutations.

Looking into the future, it is anticipated that engineered carriers will undergo further evolution and refinement, eventually becoming indispensable collaborators for CRISPR‐based therapies. With each iteration, these carriers are expected to enhance their design and functionality, enabling researchers to explore novel frontiers in precision medicine. Such advancements hold immense potential to reshape the treatment landscape, empowering researchers to precisely edit the genomic content of specific cell types within the human body. Consequently, this paves the way for a new era of targeted and personalized medicine, where therapeutic interventions are tailored to individual genetic profiles, ushering in a transformative era of healthcare. By harnessing the power of CRISPR in tandem with advanced delivery platforms, researchers can envision a future where diseases once deemed incurable can be effectively addressed at their genetic roots.

## Conflict of Interest

The authors declare no conflict of interest.

## Author Contributions

N.R., M.M.G., S.B., E.C., S.A., M.F., S.S.E., Z.M., and F.N.M.: collected data, created illustrations, and authored the manuscript. Y.Z., M.P., V.U., and E.D.: reviewed the manuscript. M.M.G., S.B., and B.R.S.: conceived and designed the study, and revised the manuscript. All authors reviewed and approved the final manuscript.
